# Valine potentiates cefoperazone-sulbactam to kill methicillin-resistant *Staphylococcus aureus*

**DOI:** 10.1128/msystems.01244-24

**Published:** 2024-12-18

**Authors:** Shao-hua Li, Yuan Tao, Zhi-cheng Yang, Huan-zhe Fu, Hui-yin Lin, Xuan-xian Peng, Hui Li

**Affiliations:** 1 State Key Laboratory of Bio-Control, School of Life Sciences, Southern Marine Science and Engineering Guangdong Laboratory (Zhuhai), Sun Yat-sen University, Guangzhou, China; 2 Laboratory for Marine Fisheries Science and Food Production Processes, Qingdao National Laboratory for Marine Science and Technology, Qingdao, China; University of California San Diego, La Jolla, California, USA

**Keywords:** valine, MRSA, cefoperazone and sulbactam, metabolic reprogramming, antibiotic resistance

## Abstract

Metabolic state-reprogramming approach was extended from Gram-negative bacteria to Gram-positive bacterium methicillin-resistant *Staphylococcus aureus* (MRSA) for identifying desired reprogramming metabolites to synergize existing antibiotic killing to MRSA. Metabolomics comparison between MRSA and methicillin-sensitive *Staphylococcus aureus* showed a depressed metabolic state in MRSA. Valine was identified as the most depressed metabolite/biomarker, and valine, leucine and isoleucine biosynthesis as the most enriched metabolic pathway. Thus, valine was used as a reprogramming metabolite to potentiate existing antibiotic killing to MRSA. Among the tested antibiotics, valine synergized cefoperazone-sulbactam (SCF) to produce the greatest killing effect. The combined effect of SCF and valine was demonstrated in clinical MRSA isolates and in mouse systemic and thigh infection models. Underlying mechanisms were attributed to valine-induced the activation of the pyruvate cycle/the TCA cycle and fatty acid biosynthesis. The activated pyruvate cycle/the TCA cycle elevated proton motive force by NADH and the activated fatty acid biosynthesis promoted membrane permeability by lauric acid. Both together increased cefoperazone uptake, which outpaces efflux action and thereby intracellular drug is elevated to effectively kill MRSA. These results provide the combination of valine and SCF to produce a new drug candidate effective against MRSA.

**IMPORTANCE:**

Methicillin-resistant *Staphylococcus aureus* (MRSA) is possibly the most infamous example of antibiotic resistance and new antibiotics are urgently needed to control it. The present study used metabolic state-reprogramming approach to identify an ideal biomarker as an antibiotic adjuvant for reversing the metabolic state of MRSA. The most repressed valine was identified as the adjuvant. Exogenous valine most effectively potentiated cefoperazone-sulbactam (SCF) to kill MRSA *in vitro* and *in vivo*. Viability of 18 clinical MRSA isolates was reduced by the top 276.64-fold in the presence of valine and SCF. In mouse models, lower bacterial load in liver, spleen, kidney, thigh, and higher survival were determined in the SCF + valine than valine or SCF alone. Valine promoted MRSA to increase SCF uptake that overcomes the efflux and enzymatic hydrolysis. It also extended the PAE of SCF. These occur because valine activates the pyruvate cycle to elevate proton motive force by NADH and increases membrane permeability by lauric acid. Therefore, the combination of valine and SCF is a new drug candidate effective against MRSA.

## INTRODUCTION

Antibiotic resistance is a big challenge during the treatment of bacterial infections due to the overuse and misuse of antibiotics. Among dangerously antibiotic-resistant bacterial pathogens, methicillin-resistant *Staphylococcus aureus* (MRSA) is possibly the most infamous example of antibiotic resistance since it is capable of becoming resistant to all classes of antibiotics clinically available (1). MRSA is not only easy to drug resistance, but also a dangerously Gram-positive pathogen. It is responsible for at least 25–50% of *S. aureus* infections in patients in contact with healthcare and is of major concern due to the high morbidity and mortality (2, 3). When traditional strategies in developing novel drugs to treat MRSA have not been very successful (4), development of new effective treatments to control MRSA infection is urgent

The recently,developed metabolic state-reprogramming approach provides an effective way to combat antibiotic-resistant pathogens. The approach is dependent upon that antibiotic-resistant and -sensitive bacteria have antibiotic-resistant and -sensitive metabolic states, respectively (5–7). Crucial biomarkers identified from the comparison between the two states can preprogram the antibiotic-resistant metabolic state into an antibiotic-sensitive metabolic state and thereby elevate bacterial sensitivity to antibiotics (7–10). The approach has been highly effective in potentiating clinically conventional antibiotics that are already resistant to kill Gram-negative pathogens (11). These include the synergistic use of glucose/alanine with aminoglycosides (kanamycin, gentamicin, and amikacin) against multidrug-resistant *Edwardsiella tarda*, cefoperazone-sulbactam (SCF)-resistant *Pseudomonas aeruginosa*, and gentamicin-resistant *Vibrio alginolyticus* (6, 12–16), of glutamine with penicillins (ampicillin) against multidrug-resistant *Escherichia coli* and their biofilm and persisters (7), and of triclosan with quinolones against *V. alginolyticus* (17, 18). In addition, metabolites are also selected directly to potentiate antibiotic killing. For example, carbon sources such as glucose potentiate aminoglycosides to kill multidrug-resistant *E. coli* (19) and amino acids such as leucine promotes sarafloxacin to kill *Salmonella* (20). The metabolic state-reprogramming approach is not adopted to combat MRSA, but it has been reported that the use of the metabolites effective against gram-negative bacteria promote antibiotics to kill MRSA (21). Rutowski et al. show that exogenous glucose in combination with a sublethal dose of penicillins (methicillin) enhances the antibiotic killing to MRSA and methicillin-sensitive *Staphylococcus aureus* (MSSA) (4). Fan et al. demonstrated that uracil and glutamine synergize aminoglycosides (gentamicin) to kill MRSA USA300 (22, 23). However, whether the metabolic reprogramming approach can be used to identify a reprogramming metabolite through comparison between MRSA and MSSA and then the reprogramming metabolite is used to reverse the resistance of MRSA for potentiating antibiotic killing is unknown. More importantly, more efficient reprogramming of metabolites can be optimized if this approach is feasible.

Here, GC-MS-based metabolomics was used to compare metabolic states between clinically isolated MRSA and MSSA. Depressed valine in MRSA was identified as a reprogramming metabolite. Exogenous valine potentiated SCF to effectively eliminate MRSA *in vitro* and *in vivo*. This finding highlights the way in identifying reprogramming metabolites and then using them to synergize antibiotics that are already resistant to kill MRSA.

## RESULTS

### Identification of MRSA and MSSA isolates

In this study, 19 MRSA and 9 MSSA strains were collected from infected patients. Since *mecA* is a specific gene carried by MRSA and *femB* and *pvl* (encoding panton-valentine leucocidin [PVL]) are two specific genes carried by *S. aureus* (2), these three genes were measured in these strains. Note that *pvl* also distinguishes community-acquired MRSA from hospital-acquired MRSA (24). *femB* was detected in all MRSA and MSSA isolates, while *pvl* was detected in eight MSSA strains and all MRSA, and *mecA* was determined only in MRSA isolates (Fig. S1; Table S1). Furthermore, minimum inhibitory concentration (MIC) to daptomycin, vancomycin, benzoxiline, and SCF was measured based on Clinical and Laboratory Standards Institute (CLSI) (CLSI 2022). All MRSA strains were resistant to oxacillin, while MSSA strains were sensitive to oxacillin. No vancomycin-resistant bacteria were found in these strains. (Table S2). These results support the identification of these MRSA and MSSA strains.

### Identification of β-lactamases

β-Lactamases possess a high ability to hydrolyze β-lactams. β-Lactamases, including carbapenemases (CREs), extended-spectrum β-lactamases (ESBLs), and AmpC, were measured in the 19 MRSA isolates. Among them, 16 (84.2%) CREs, 16 (84.2%) ESBLs, and 15 (78.9%) AmpC were positive. Specifically, 16 isolates were positive, 2 isolates were negative, 1 isolate was positive only for CRE, and 2 isolates were double-positive for CRE and ESBL and double-positive for ESBLs and AmpC, respectively (Table S3). These results indicate that β-lactamases are contained in almost all of these MRSA tested.

### Metabolic profiles of MSSA and MRSA

**Fig 1:**
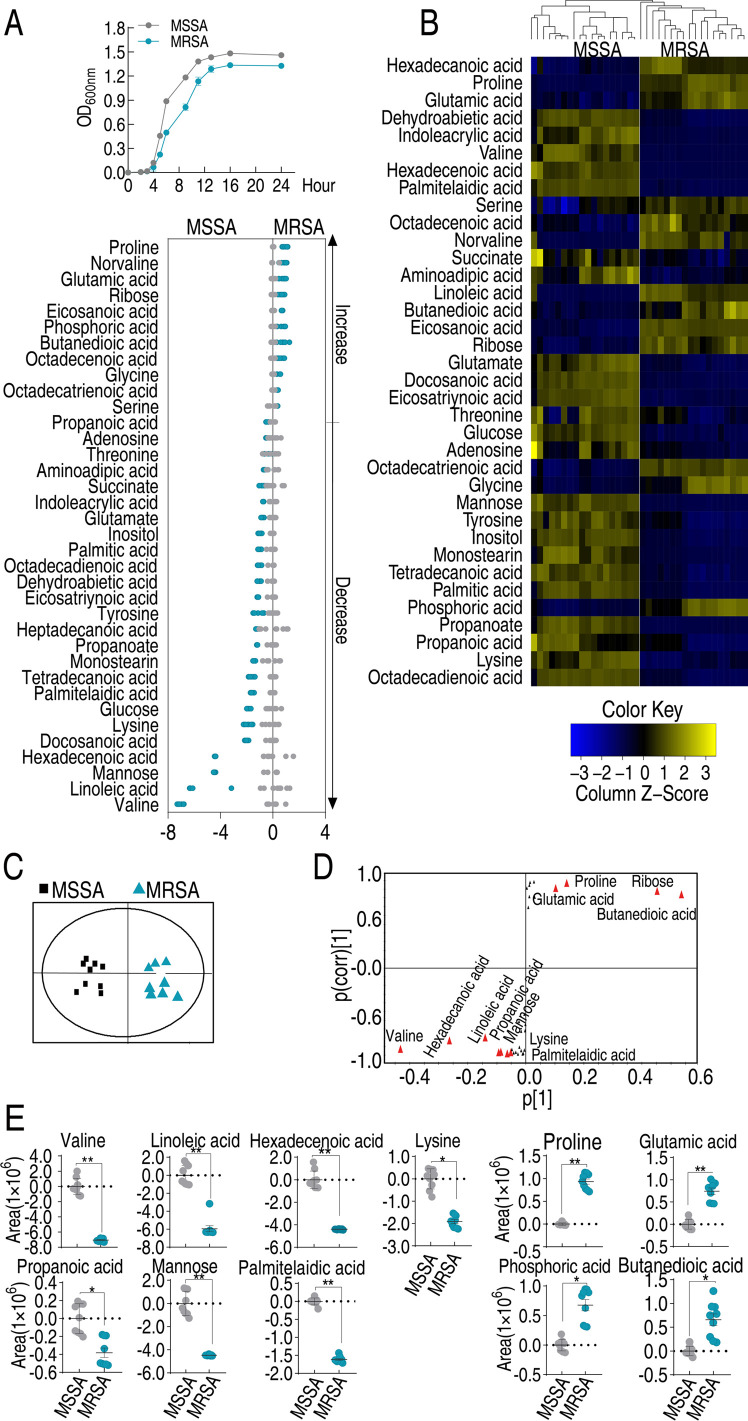
Differential growth curve and metabolome between MSSA and MRSA. (**A**) Growth curve of five MSSA isolates (MSSA1–5) and five MRSA isolates (MRSA1–5). (**B**) Heat maps of differential abundance of metabolites (row) from supernatant of whole-cell lysates using R language. Yellow and blue indicate the increase and decrease of the metabolites relative to the mean and SD of the row metabolite level, respectively (see color scale). The differential abundance of metabolites was determined by a two-sided Mann–Whitney *U* test coupled with a permutation test using SPSS 17.0 software. (**C**) *Z*-score plots of differential metabolites of MRSA based on MSSA. The *Z*-score was calculated using the formula *Z* = (*M* − *y*)/MSE, where *M* is the measured value, *y* is the mean, and MSE is the standard error. Data from MRSA are separately scaled to the mean and SD of MSSA. Each point represents one metabolite in one technical repeat and is colored by sample types. (**D**) OPLS-DA analysis of MSSA and MRSA using SIMCA + 12.0. Each dot represents one technical replicate. (**E**) S-Plot generated by OPLS-DA identifies differential metabolites based on *t*[1] in panel **D**. Each triangle represents individual metabolite, where potential biomarkers are highlighted with red, which is greater or equal to 0.05 and 0.5 for absolute value of covariance *P* and correlation *P*(corr), respectively. Otherwise, triangle is marked black color. (**F**) Relative abundance of candidate biomarkers from panel **E**. Results are displayed as mean ± SD of four biological replicas and significant differences are identified by Kruskal–Wallis test (**P < 0.01).

Recent reports have indicated that the bacterial metabolic state is responsible for antibiotic resistance (6, 10, 25, 26). To determine the metabolic difference between MRSA and MSSA strains, first, growth curve was measured in five MRSA and five MSSA isolates. Lower growth was detected in MRSA than in MSSA (Fig. 1A). Then, gas chromatography-mass spectrometry (GC-MS) based metabolomics was performed in nine MRSA and nine MSSA isolates with two technical replicates for each isolate, yielding a 36 data set. The reproducibility coefficients of the combined technical replicates, as determined through correlation coefficient analysis, ranged from 0.986 to 0.992, indicating a high level of reproducibility in the GC-MS data (Fig. S2A). A total of 78 metabolites were identified and classified into 26% carbohydrate, 23% amino acids, 9% nucleotide, 29% lipid, and 13% others (Fig. S2B). A heat map of metabolite abundance was constructed (Fig. S2C). MRSA and MSSA strains were clustered independently, indicating that the metabolic state is related to a physiological difference between MRSA and MSSR. The differential abundance of metabolites was determined by a two-sided Mann–Whitney *U* test coupled with a permutation test using SPSS 17.0 software. A total of 36 metabolites were determined with significant differences (*P* < 0.05) (Fig. 1B). Among them, 11 were upregulated and 25 were downregulated, where valine was the most repressed metabolite. The deviations of these differential metabolites were exhibited by *Z*-value, where valine was the most downregulated metabolite (Fig. 1C). To identify crucial metabolites involved in drug resistance, orthogonal partial least squares discriminant analysis (OPLS-DA) was employed and patterns in the samples were determined, component *t*[1] distinguished MRSA from MSSA (Fig. 1D), which was consistent with the above heat map clustering results. Metabolites with more significant variability [covariance *P* and correlation *P*(corr) values greater than or equal to 0.05 and 0.5, respectively] were further identified by plotting S-plots, and a total of 13 biomarkers were identified in this analysis, of which 7 (valine, linoleic acid, hexadecenoic acid, propenoic acid, mannose, lysine, and palmitelaidic acid) were downregulated and 4 (butanedioic acid, ribose, proline, and glutamic acid) were upregulated (Fig. 1E). Among the seven repressed metabolites, valine showed the most significant variability. The scatter plot of the relative abundance of metabolites is shown in Fig. 1F. These results indicate that the repressed valine is the most metabolic characteristic feature in MRSA.

### Enrichment of metabolic pathways involved in MRSA resistance

**Fig 2:**
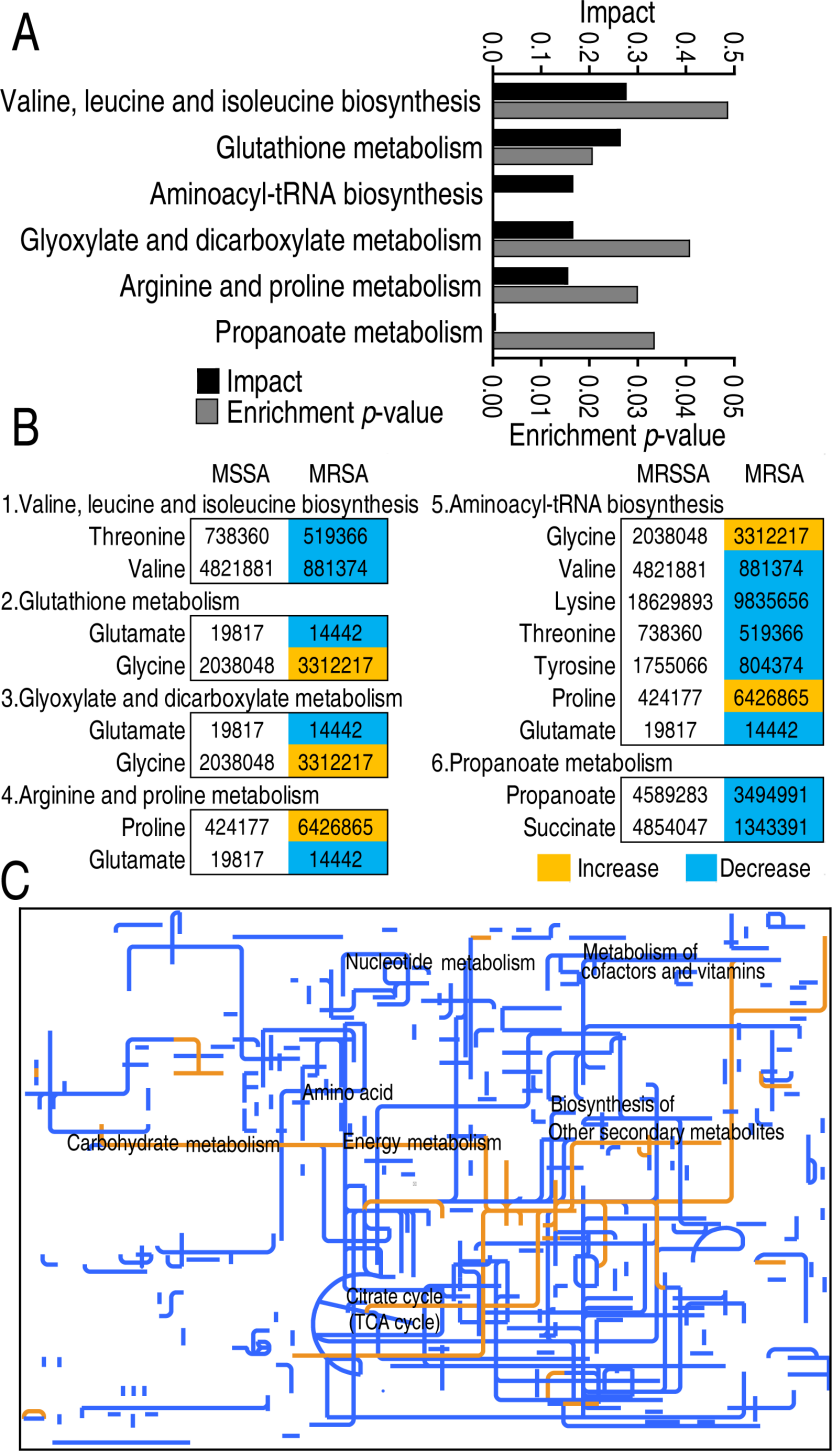
Pathway analysis of differential metabolites. (**A**) Enriched metabolic pathways were obtained by the website analysis (http://www.metaboanalyst.ca/.) (**B**) Metabolite abundance changes with the color blocks in enriched pathways. (**C**) Metabolic network pathways analysis of differential abundances of metabolites between MRSA and MRSA by iPath2.0 (https://pathways.embl.de/). Yellow and blue lines indicate upregulation and downregulation of metabolic pathways, respectively.

To know the most affected metabolic pathways in MRSA, MetaboAnalyst 4.0 was employed to examine the metabolic pathways associated with drug resistance, specifically focusing on the enrichment of six metabolic pathways (*P* < 0.05). Among the six metabolic pathways, valine, leucine, and isoleucine biosynthesis had the greatest impact on the drug resistance (Fig. 2A). All (valine, leucine, and isoleucine biosynthesis; propanoate metabolism), almost (aminoacyl-tRNA biosynthesis), or half (glutathione metabolism; glyoxylate and dicarboxylate metabolism; arginine and proline metabolism) of the metabolites in the six metabolic pathways were decreased (Fig. 2B), suggesting that the enriched metabolic pathways are repressed. iPath analysis provides an interactive map for visualizing metabolic pathways. It was shown that metabolic repression is dominant in MRSA (Fig. 2C). Taken together, MRSA exhibited a globally repressed metabolism, where the top repressed valine is included in the top enriched metabolic pathway.

### Exogenous valine reverses MRSA resistance to SCF

**Fig 3:**
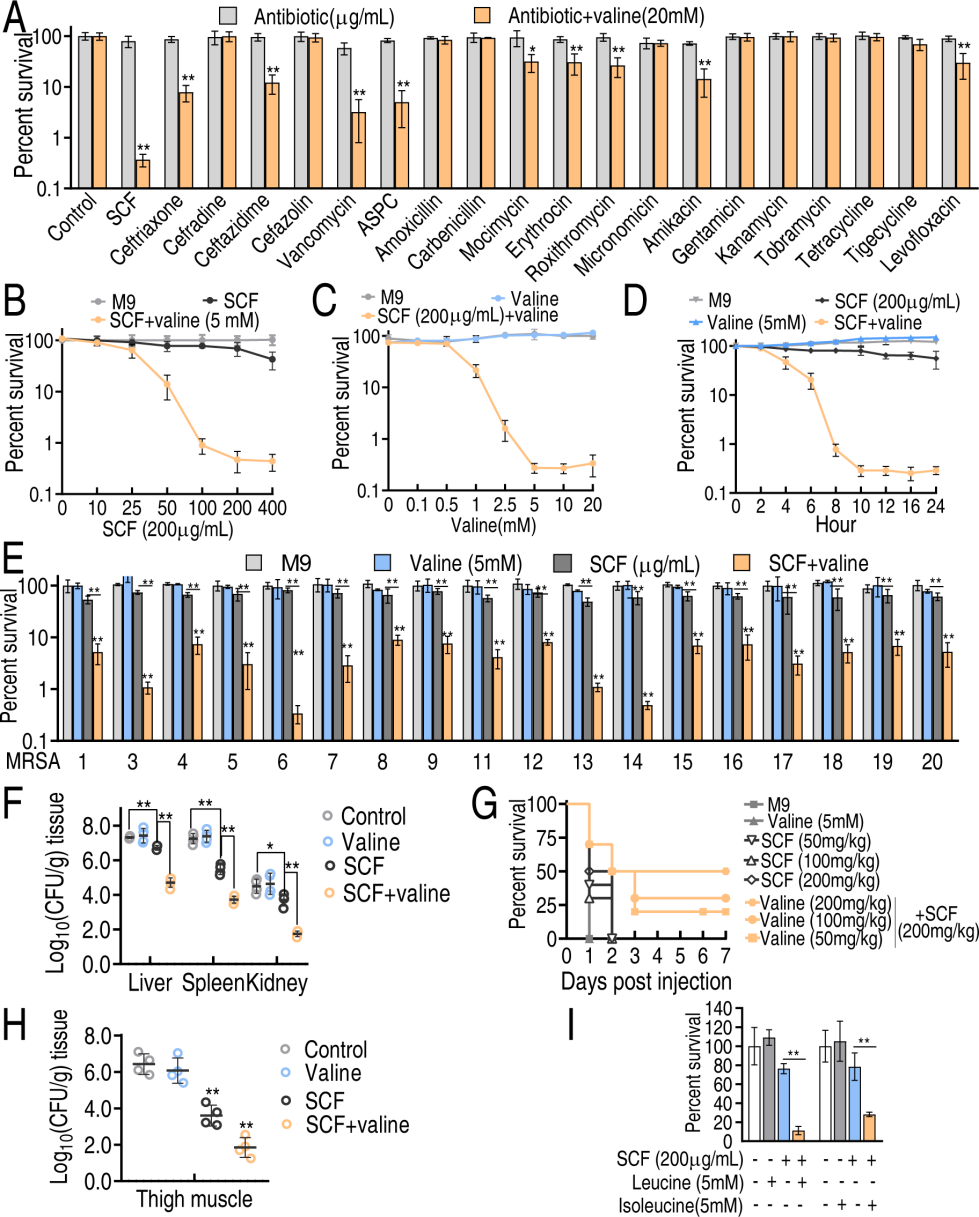
Antibacterial efficacy by SCF in the absence or presence of valine. (**A**) Percent survival of MRSA2 in the presence of the indicated antibiotics with or without 5 mM valine. SCF, cefoperazone sodium and sulbactam sodium; ASPC, amoxicillin sodium clavulanate potassium. Antibiotic doses are as follows: 200 µg/mL for SCF; 300 µg/mL for ceftriaxone, cefradine, ceftazidime, cefazolin; 150 µg/mL for vancomycin; and 400 µg/mL for the others. (**B**) Percent survival of MRSA2 in the absence or presence of the indicated concentrations of SCF plus 5 mM valine. (**C**) Percent survival of MRSA2 in the absence or presence of the indicated concentrations of valine plus 200 µg/mL SCF. (**D**) Percent survival of MRSA2 in the absence or presence of the indicated incubation time with 5 mM valine and 200 µg/mL SCF. (**E**) Percent survival of 18 MRSA isolates (2.5 × 10^7^ CFU/mL) in the absence or presence of 5 mM valine, 200 µg/mL SCF, and both. (**F**) Bacterial load of liver, kidney, and spleen of mice infected with MRSA2. Mice were intraperitoneally infected with 2.5 × 10^6^ CFU of MRSA2 and divided into four groups, four male Balb/c mice per group. The four groups were separately intravenously injected with saline solution, 200 mg/kg SCF, 200 mg/kg valine, or both in an hour later. Spleen, kidney, and liver of these treated mice were collected at 24 h and homogenized for spot plate counting. (**G**) Percent survival of mice in the absence or presence of the indicated dose of SCF or/and the indicated dose of valine. Mice were intraperitoneally infected with 2.5 × 10^7^ CFU of MRSA2 and divided into eight (sub)groups to be treated as the indicated post 1 h of infection, 10 mice per group. Survival of mice was monitored for 7 days. (**H**) Bacterial load of mouse thigh infected with MRSA2. Mice were intramuscularly infected with 2.8 × 10^5^ CFU of MRSA2 and divided into four groups. The four groups were separately treated with saline solution, 200 mg/kg SCF, 200 mg/kg valine, or both post 2 h infection. The thigh muscle infected was collected at 26 h and homogenized for spot plate counting. (**I**) Percent survival of MRSA2 in the absence or presence of 5 mM leucine or isoleucine plus 200 µg/mL SCF. Data are mean ± SD from three biological replicates. *, *P* < 0.05; **, *P* < 0.01.

Replenishment of the repressed crucial biomarker can reverse antibiotic resistance (7, 27). Thus, we hypothesized that valine replenishment boosts the top repressed metabolic pathway to promote MRSA sensitivity to antibiotics. To test this idea, 20 drugs from seven classes of antibiotics were used to explore the synergistic effect with valine to MRSA2. Among them, SCF, ceftriaxone, ceftazidime (CAZ) (cephalosporins), vancomycin (glycopeptides), amoxicillin sodium clavulanate potassium (ASPC) (penicillins), mocimycin, erythrosine, roxithromycin, amikacin (aminoglycosides), and levofloxacin (fluoroquinolones) showed the synergistic killing with valine with increasing 2.81- to 215.7-fold. Among them, SCF was the best one with 215.7-fold elevation (Fig. 3A). Thus, SCF was selected to carefully examine the optimal conditions for the best synergistic efficacy to MRSA2. Viability was reduced with the increasing valine, where the synergistic efficacy appeared in the presence of 50 µg/mL SCF and arrived at the top of 200 µg/mL SCF (Fig. 3B). Further experiments showed that the synergy of 5 mM valine with 200 µg/mL SCF in 10 h incubation led to the best effect (Fig. 3C and D). When the optimal killing conditions were adopted in 19 MRSA isolates, lower viability was detected in the synergistic use of SCF and valine than either SCF or valine alone with increasing 7.30- to 315.4-fold (Fig. 3E). Therefore, the synergistic use is effective to these MRSA isolates.

To further test whether valine-potentiated SCF killing was implemented in mice, systemic and thigh infections were performed. In systemic infection, plate counting of organ bacteria and mouse survival experiments were adopted. Lower bacterial number in liver, spleen, and kidney was measured in the synergistic use of SCF and valine than valine or SCF alone (Fig. 3F). Survival of mice was elevated in the combination of valine and SCF compared to SCF alone. Specifically, all mice died in the presence of 50–200 mg SCF/kg, whereas 20%, 30%, and 50% survival were found in the synergy of 50, 100, and 200 mg/kg valine with 200 mg/kg SCF, respectively (Fig. 3G). Similarly, lower ability was measured in the synergy than SCF alone in the thigh model (Fig. 3H). Therefore, the valine-potentiated SCF killing is effective *in vivo*. In addition, we also tested whether other branched-chain amino acids had the potential. Viability of MRSA2 was reduced by 2.7-fold and 7.1-fold in the presence of isoleucine or leucine, respectively (Fig. 3I), but the elevated fold was much lower than that promoted by valine (215.7-fold). Therefore valine was used for the following experiments.

### Valine promotes SCF uptake and influences post-antibiotic effect of SCF

**Fig 4:**
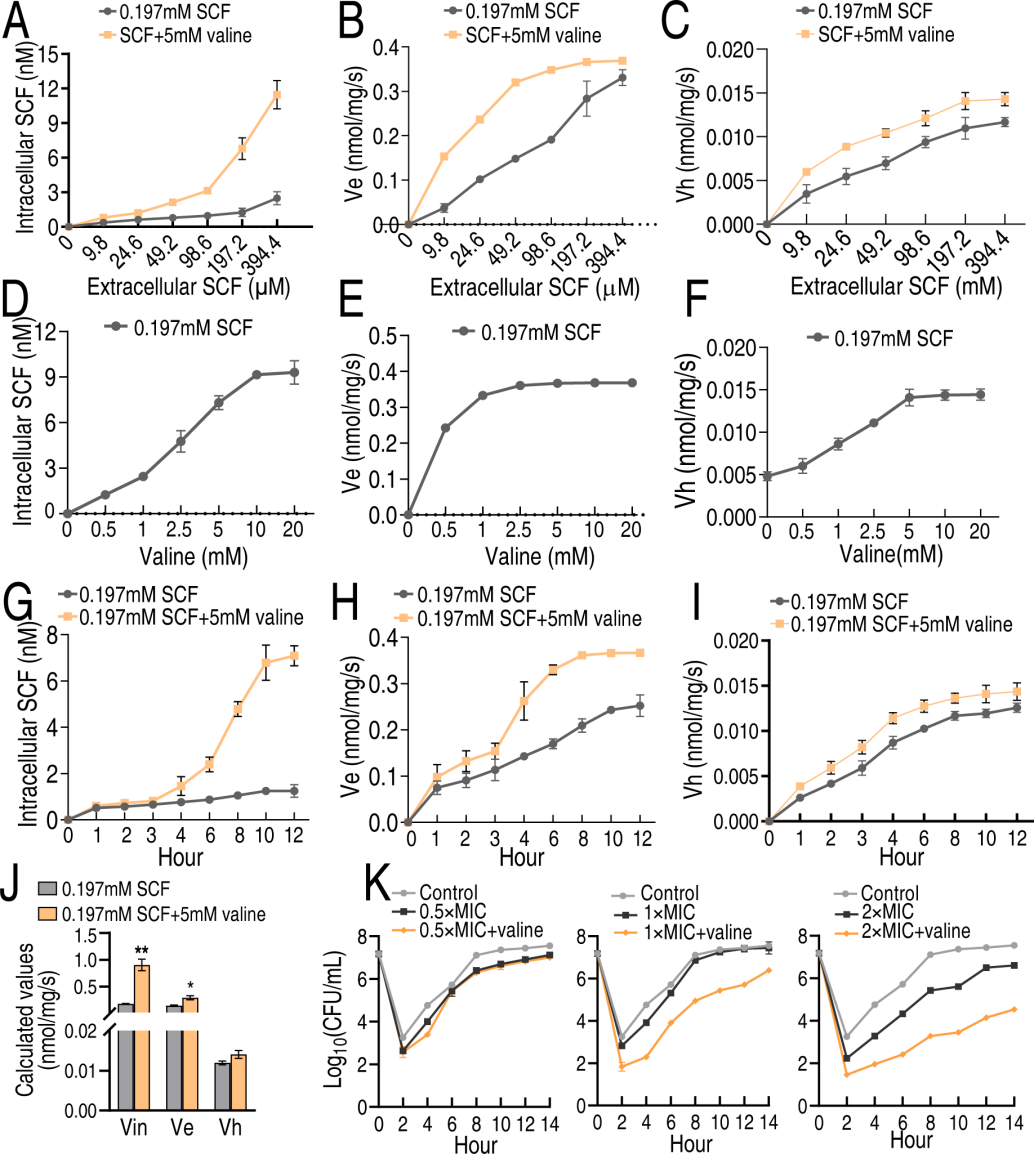
Valine promotes MRSA2 to increase the uptake of SCF. (**A–C**) Relationship of increasing extracellular drug concentrations with influx (**A**), efflux (**B**), and enzymatic hydrolysis (**C**) in the presence or absence of 5 mM valine and the indicated concentration of SCF. Note that 9.8, 24.6, 49.2, 98.6, 197.2, and 394.4 µM SCF are equal to 10, 25, 50, 100, 200, and 400 µg/mL SCF, respectively. (**D–F**) Relationship of increasing extracellular valine concentrations with influx (**D**), efflux (**E**), and enzymatic hydrolysis (**F**) in the presence of 0.197 mM SCF. (**G–I**) Relationship of increasing incubation periods with influx (**G**), efflux (**H**), and enzymatic hydrolysis (**I**) in the presence of 0.197 mM SCF with or without 5 mM valine. (**J**) Calculated values of *V*_in_ and *V*_e_ in MRSA2 at *C*_0_ = MIC. (**K**) Effect of exogenous valine (5 mM) on PAE at different multiplicity of MICs. Data are mean ± SD from three biological replicates.

We supposed that the valine-potentiated killing is related to the change in intracellular drug concentration due to the high efficiency in the presence of valine. To explore this, a dynamic observation on SCF with and without valine was carried out to test whether the elevated SCF uptake outpaces the SCF dampening by efflux and enzymatic hydrolysis in MRSA2, which producing CREs, ESBLs, and AmpC (Table S3). The SCF concentration, valine concentration, and incubation time were used as the same as Fig. 3B through D, where SCF was expressed in terms of concentration rather than mass based on the pharmacokinetic analysis. Valine promoted more drug uptake, efflux, and hydrolysis with the increasing extracellular SCF, but the increased uptake was higher than the efflux and hydrolysis (Fig. 4A through C). Then, the intracellular concentration of SCF was measured in the presence of different concentrations of valine. Intracellular SCF concentration was increased with increasing concentrations of valine plus 0.197 mM SCF (Fig. 4D). Under the same condition, efflux and hydrolysis were increased but lower than the uptake (Fig. 4E and F). Furthermore, a time-dependent uptake, efflux, and hydrolysis were performed in the absence or presence of valine. The uptake was increased with increasing incubation time and arrived at the top in 10 h (Fig. 4G), while the efflux and hydrolysis were elevated with the time in medium with and without valine (Fig. 4H and I). However, exogenous valine caused more uptake that outpaced the dampening by the valine-promoted efflux and hydrolysis. (Fig. 4G through I). Collectively, under MIC concentration, exogenous valine increased drug efflux to 0.90 nmol/mg per s, which leads to the equation of *V*_in_ ≈ 3.05 (*V*_e_) (Fig. 4J). Therefore, exogenous valine promotes intracellular SCF concentration to kill MRSA.

Post-antibiotic effect (PAE) is a characteristic of pharmacodynamics. It is defined as the phenomenon in which a drug continues to inhibit bacterial growth after bacteria have been briefly exposed to an antibiotic (28). Thus, an understanding whether the synergistic use of SCF and valine changed the PAE of SCF may reveal one more mechanism of valine-potentiated SCF killing. To do this, MRSA2 was used as the experimental strain to study the PAE of SCF in the presence or absence of valine. The PAE was 1.36 ± 0.04 h at 0.5 × MIC, 1.60 ± 0.03 h at 1 × MIC, and 1.94 ± 0.03 h at 2 × MIC in SCF alone. However, 1.69 ± 0.02 h at 0.5 × MIC, 2.17 ± 0.04 h at 1 × MIC, and 2.66 ± 0.01 h at 2 × MIC were measured in the presence of the combination of valine with SCF (Fig. 4K). These results indicate that valine extends the PAE of SCF.

### Valine reprograms MRSA metabolic state

**Fig 5:**
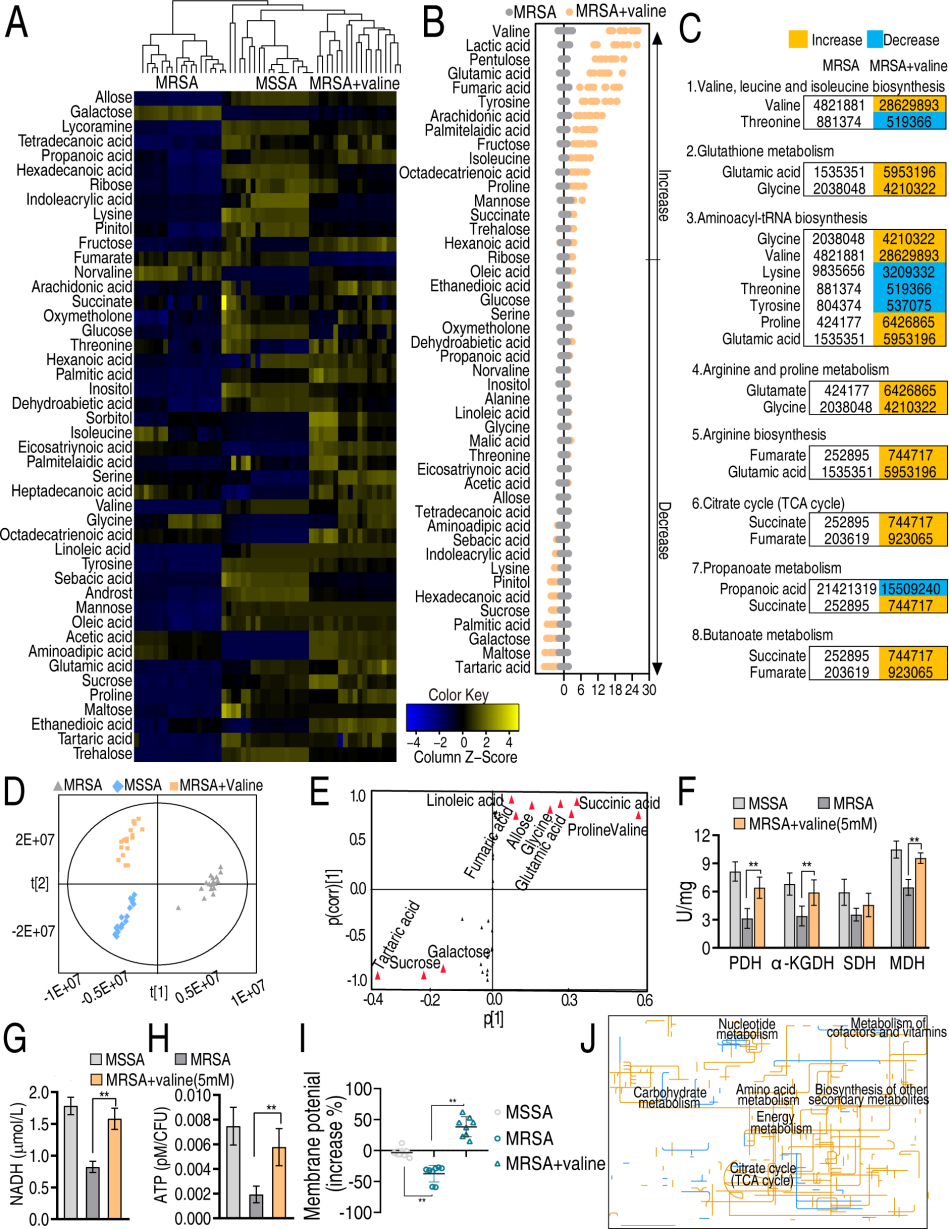
Metabolic reprogramming by valine in MRSA. (**A**) Heatmap analysis of absolute differential metabolites from supernatant of whole-cell lysates among MSSA, MRSA, and MRSA + valine using R language. (**B**) *Z*-Plot analysis of differential metabolites from MRSA + valine compared to MRSA. (**C**) Enriched metabolic pathways and metabolite abundance in these enriched metabolic pathways in the absence or presence of valine. Yellow and blue lines indicate upregulation and downregulation of metabolic pathways, respectively. (**D**) OPLS-DA analysis among MSSA, MRSA, and MRSA + valine. Each dot represents the technique replicated in the plot. (**E**) S-plot generated by OPLS-DA. Triangle represents individual metabolite, where potential biomarkers are highlighted with red, which is greater or equal to 0.05 and 0.5 for absolute value of covariance *P* and correlation *P*(corr), respectively. Otherwise, triangle is marked black color. (**F–I**) Activity of the indicated enzyme (**F**) and level of NADH (**G**), ATP (**H**), and PMF (**I**) in MSSA, MRSA, and MRSA + valine. (J) Metabolic network pathways analysis of differential abundances of metabolites between MRSA and MRSA + valine by iPath2.0 (https://pathways.embl.de/). Yellow line represents increase in MRSA + valine but decrease in MRSA; blue line represents decrease in MRSA + valine but increase in MRSA. Data are mean ± SD from three biological replicates. *, *P* < 0.05; **, *P* < 0.01.

Key biomarkers reprogram antibiotic-resistant metabolic states into antibiotic-sensitive ones, thereby being susceptible to antibiotics that are already resistant (7, 29). To know what metabolic state of MRSA is reprogrammed by valine, GC-MS-based metabolomics was utilized to measure the valine-reprogrammed metabolome in MRSA, and MRSA without valine and MSSA were used as controls. A total of 79 metabolites were quantified in each of the three groups. Cluster analysis showed that MRSA without valine was separated from MSSA and MRSA + valine (Fig. S3A), suggesting that MRSA + valine had a metabolic state that was closer to MSSA than MRSA. Differential metabolites were identified using Kruskal-Wallis test (*P* < 0.01) (Fig. 5A). A total of 36 and 47 differential abundances of metabolites were identified in MSSA and MRSA + valine, respectively, compared to MRSA, where 26 were overlapped between MSSA and MRSA + valine (Fig. S3B). Based on KEGG annotation and NCBI PubChem, 47 metabolites were categorized into five groups, namely carbohydrate (26%), amino acid (26%), fatty acid (24%), nucleotide (9%), and others (15%) (Fig. S3C). *Z*-Score analysis was based on the sample value minus the mean and divided by the standard deviation. Among the 47 differential abundances of metabolites in MRSA + valine, 17 were upregulated and 30 were downregulated (Fig. 5B). Pathway enrichment analysis showed that eight metabolic pathways are enriched. Among them, five (glutathione metabolism, arginine and proline metabolism, arginine biosynthesis, the TCA cycle, and butanoate metabolism) exhibited the upregulation of all metabolites (Fig. 5C). Subsequently, this study identified the sample patterns by OPLS-DA to obtain component *t*[1] and component *t*[2]. Component *t*[1] separated MSSA and MRSA + valine from MRSA and component *t*[2] separated MRSA from MRSA + valine (Fig. 5D). S-plot was generated to visualize key metabolites of the metabolic shifts. Among the 46 metabolites, 11 were identified as biomarkers based on absolute values of covariance *P* and correlation *P* (corr) with ≥0.05 and ≥0.5, respectively. Out of 11 biomarkers, 8 were elevated. They were valine, succinic acid, proline, glutamic acid, glycine, allose, fumaric acid, and linoleic acid (Fig. 5E; Fig. S4). Importantly, four elevated metabolites (glutamate, glycine, fumarate, and succinate) were included in the five enriched metabolic pathways. Among them, fumarate and succinate of the pyruvate/TCA cycle are biomarkers, suggesting that the pyruvate/TCA cycle was activated. Note that the pyruvate cycle (the P cycle) links the phosphoenolpyruvate (PEP)-pyruvate-AcCoA pathway to the TCA cycle to provide respiratory energy in bacteria (30). To demonstrate this, activity of pyruvate dehydrogenase (PDH), α-ketoglutarate dehydrogenase (α-KGDH), succinate dehydrogenase (SDH), and malate dehydrogenase (MDH) in the cycle, NADH, ATP, and proton motive force (PMF) were measured in the presence or absence of valine. Exogenous valine promoted the activity of PDH, α-KGDH, SDH, and MDH of MRSA to be close to that of these enzymes of MSSA (Fig. 5F). Lower NADH, ATP, and PMF were detected in MRSA than in MSSA, which was reversed by exogenous valine (Fig. 5G through I). iPath analysis supported that exogenous valine boosts almost of metabolic pathways, resulting in an enhanced global metabolic state (Fig. 5J).

### Exogenous valine stimulates metabolic fluxes using non-targeted tracer fate detection

**Fig 6:**
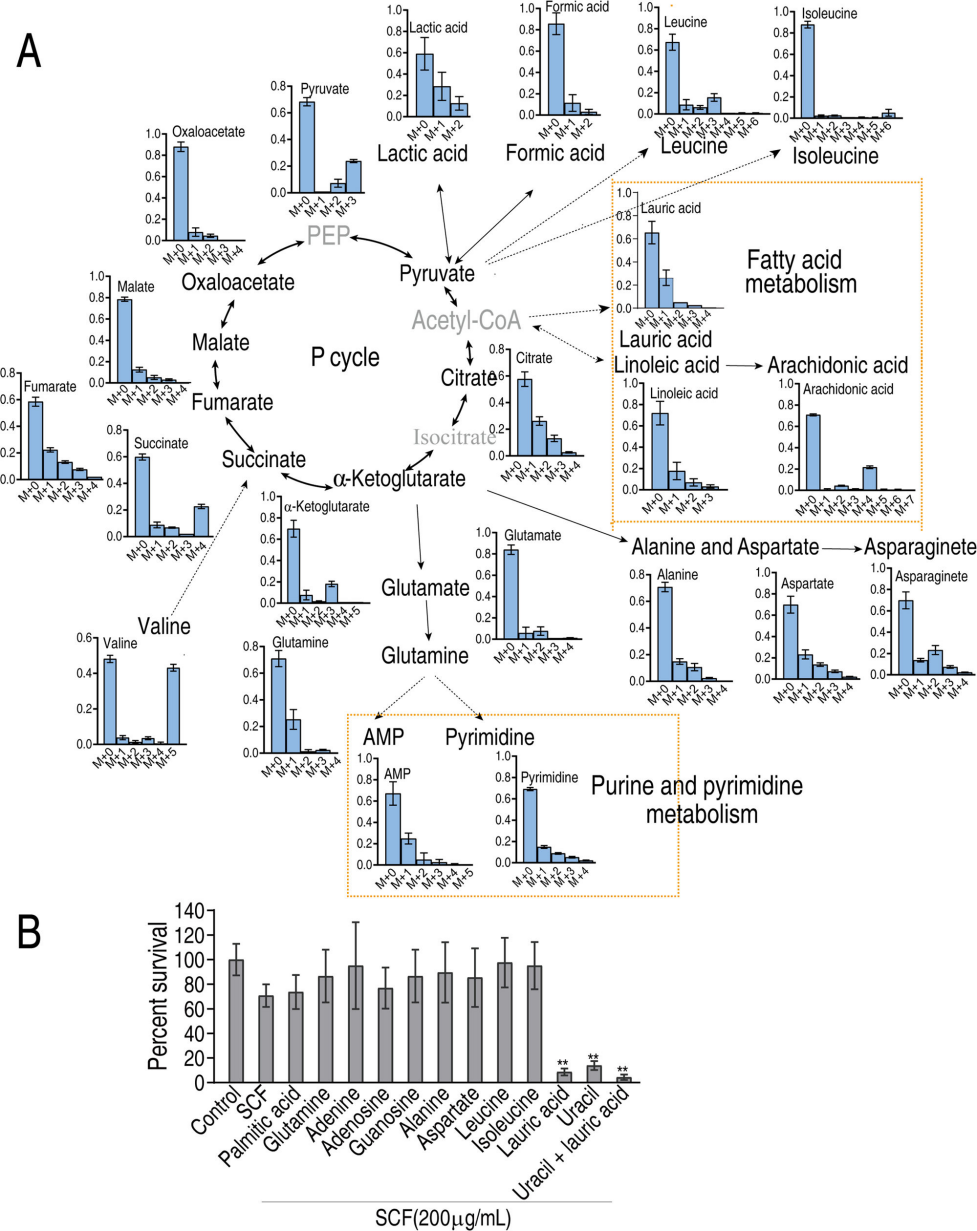
Metabolic flow of valine in MRSA2. (**A**) Mass isotopomer distributions for 13C^5^-labeled valine by non-target metabolomics. (**B**) Percent survival of MRSA2 in the absence or presence of the indicated metabolites (5 mM each), 200 µg/mL SCF or both. Data are mean ± SEM from three biological replicates. *, *P* < 0.05; **, *P* < 0.01.

We further explored metabolic fluxes stimulated by exogenous valine using non-targeted tracer fate detection (NTFD). To do this, 13C^5^-valine tracer experiment was carried out in MRSA2 by GC-MS. Mass isotopomer distributions for 13^C^ labeled valine were mainly detected in the pyruvate cycle/the TCA cycle, fatty acid biosynthesis, and purine and pyrimidine biosynthesis (Fig. 6A). To test which metabolic pathways play a role in the valine-induced potentiation, replacement of valine with plamitic acid, glutamine, adenine, adenosine, guanosine, alanine, aspartate, leucine, isoleucine, lauric acid, or uracil of these metabolic pathways to measure the viability of MRSA2 in the presence of SCF. Among them, uracil and lauric acid had the potentiation (Fig. 6B). Similar viability was detected when the synergy of uracil and lauric acid compared to lauric acid alone (Fig. 6B). These results indicate that lauric acid plays a more key role in the valine-induced potentiation.

### Mechanisms of the valine-induced potentiation

**Fig 7:**
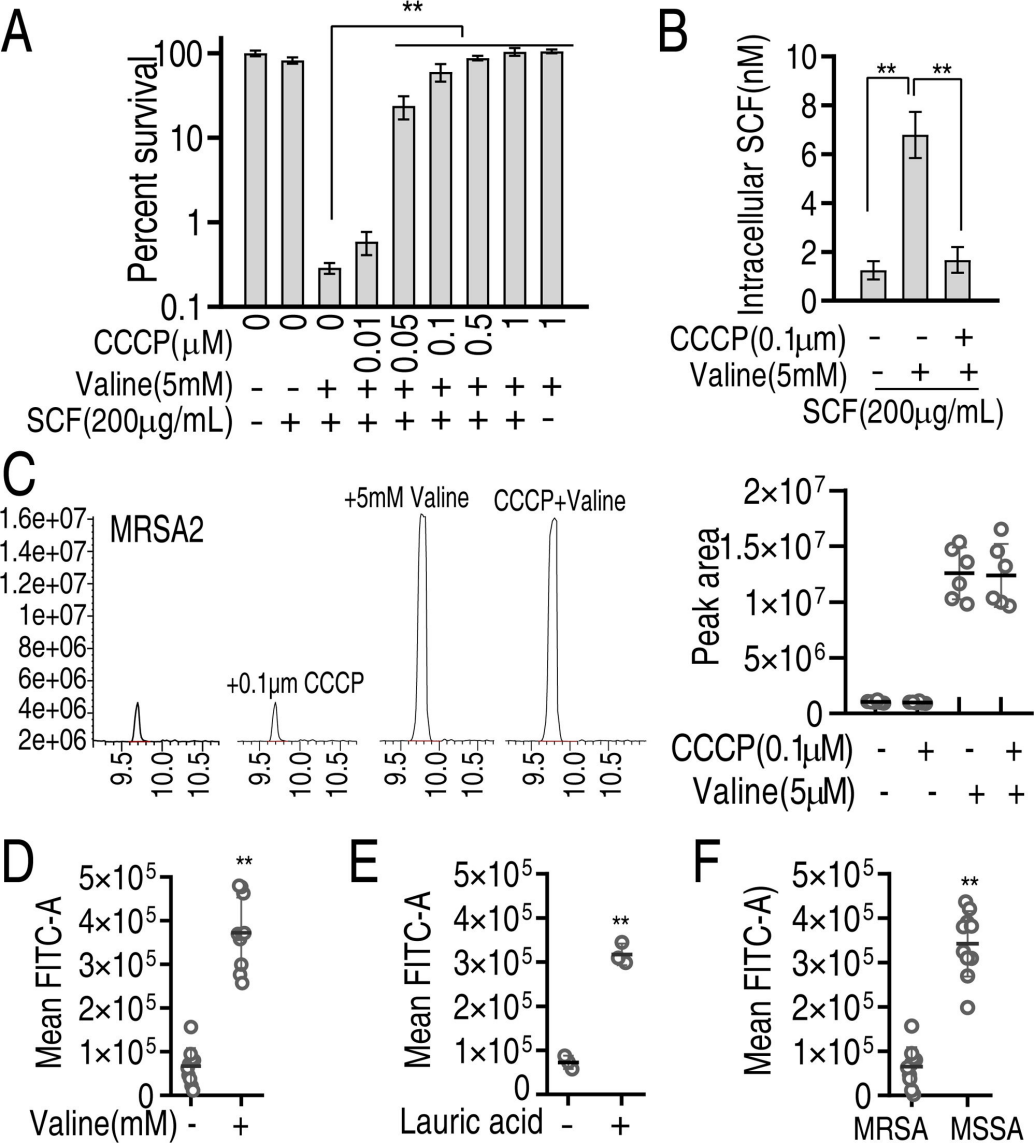
Mechanisms related to PMF and membrane permeability. (**A**) Percent survival of MRSA2 in the presence of 5 mM valine and the indicated concentrations of CCCP plus 200 mg/mL SCF. (**B**) Intracellular concentration of SCF in the presence or absence of valine or/and CCCP plus 200 mg/mL SCF. (**C**) GC-MS analysis for valine abundance of MRSA2 in the absence and presence of CCCP plus 5 mM valine. Left, GC-MS raw data; right, calculation based on data (left). (**D**) Membrane permeability of MRSA in the absence or presence of 5 mM valine. (**E**) Membrane permeability of MRSA2 in the absence or presence of 5 mM lauric acid. (**F**) Membrane permeability of MRSA and MSSA. Data are mean ± SEM from three biological replicates. **, *P* < 0.01.

The above results indicate that valine reprogramming activates the P cycle/the TCA cycle and fatty acid biosynthesis to increase NADH and lauric acid, respectively. NADH promotes PMF. Aminoglycoside uptake is PMF-dependent, but whether the SCF uptake is related to PMF is unknown. To explore this, chloro carbonyl cyanide phenyl hydrazine (CCCP), a well-known protonophore for dissipating PMF, was used to test whether it blocked the PMF-dependent SCF killing. Viability of MRSA2 was increased with the increasing CCCP dose (Fig. 7A), indicating that valine-potentiated killing was deprived by CCCP. Consistently, intracellular drug was reduced in the presence of CCCP (Fig. 7B). Note that similar valine abundance of MRSA2 was detected in the absence and presence of CCCP with or without valine (Fig. 7C), suggesting that CCCP did not affect valine transportation. Lauric acid is related to membrane permeability (31, 32). Thus, membrane permeability was measured in the absence or presence of lauric acid or valine. Lauric acid promoted the membrane permeability of MRSA2 as similar to valine (Fig. 7C and D). Consistently, lower membrane permeability of MRSA isolates was detected compared to that of MSSA isolates (Fig. 7E). In addition, CCCP did not affect the membrane permeability of MRSA2 (Fig. S5). These results indicate that the elevation of PMF and membrane permeability plays a key role in increasing intracellular drug concentration.

## DISCUSSION

**Fig 8:**
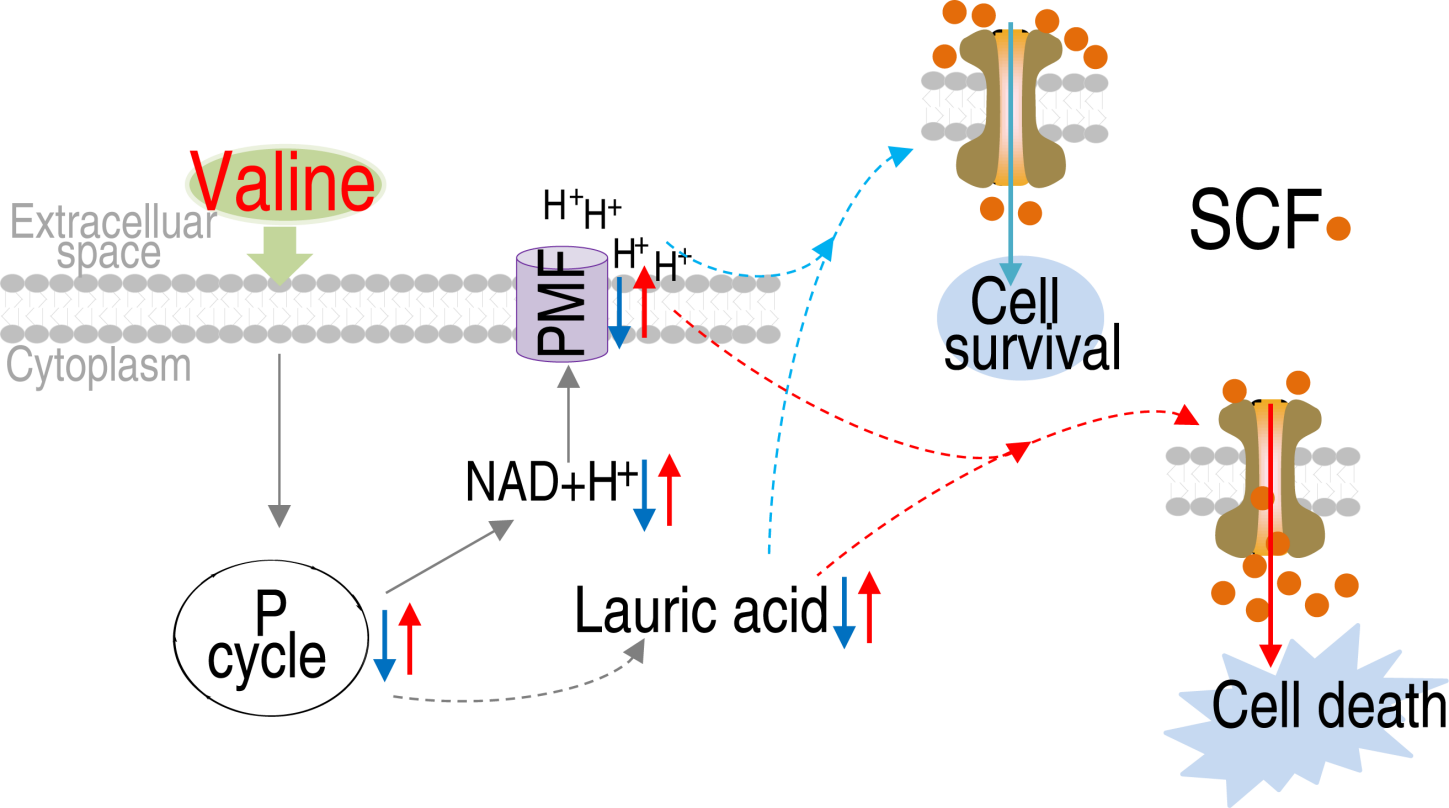
Proposed mechanism of valine-potentiated killing. MRSA exhibits a repressed metabolic state with the inactivation of the P cycle as the most characteristic feature. The inactivated P cycle causes reduced NADH and PMF as well as repressed membrane permeability, thereby decreasing drug uptake. However, exogenous valine reprograms the antibiotic-resistant metabolic state into an antibiotic-sensitive metabolic state, which promotes drug uptake through activating the P cycle/the TCA cycle to produce more NADH for elevating PMF and to generate more lauric acid for increasing membrane permeability.

The metabolic state-reprogramming has been successfully demonstrated in potentiating antibiotic killing to antibiotic-resistant pathogens and enhancing host anti-infective immunity (7, 33–35). However, potentiating antibiotic killing is not implemented in combating MRSA. MRSA is a notorious Gram-positive pathogen that has the ability to acquire resistance to most antibiotics (2, 36). Here, the comparison of global metabolic states between MRSA and MSSA leads to the identification of valine as a reprogramming metabolite to reverse the resistance of MRSA. This is because valine is the most repressed abundance of metabolite and the most crucial biomarker with the decreasing abundance. Moreover, valine, leucine, and isoleucine biosynthesis are the most enriched metabolic pathway. These also indicate that valine is repressed in the transition from MSSA to MRSA. Valine replenishment makes MRSA sensitivity to SCF due to the PMF elevation by NADH and the membrane permeability increase by lauric acid to promote drug uptake that overcomes the action of efflux pumping and β-lactamase hydrolysis (Fig. 8). Therefore, the metabolic state reprogramming approach can be used to identify the crucial biomarker as a reprogramming metabolite to reverse antibiotic resistance of MRSA.

Valine is a branched-chain amino acid. It is an important nutrient for the growth of *S. aureus* and the most abundant amino acid that is significantly released into the media at the stationary phase of *S. aureus* (37, 38). Valine together with other branching amino acids leucine and isoleucine is a corepressor for CodY, which regulates virulence gene expression. Note that there is a big difference in the potential between valine and leucine; isoleucine suggests that valine has unique mechanisms to potentiate SCF killing. Thus, valine plays a role as an important link between the metabolic state and virulence of the cell (37). This may be used to explain why virulence decreases with elevated antibiotic resistance in MRSA (39). The undirected profiles of metabolites by liquid chromatography and hydrophilic interaction in six CC5-MRSA exhibit higher abundant valine in vancomycin-intermediate *S. aureus* than heterogeneous vancomycin-intermediate *S. aureus* (40). However, whether valine is a biomarker identifying MRSA from MSSA and promoting antibiotic killing to MRSA is unknown. Here, valine is identified as the most repressed metabolite in MRSA isolates compared to MSSA isolates. More interestingly, valine replenishment reverses MRSA resistance and elevates SCF killing to MRSA *in vitro* and *in vivo*. Therefore, the synergistic effect of valine and SCF indicates that the combination is expected to produce a new effective drug against MRSA.

Recent reports have shown that the synergistic effect of a metabolite with an antibiotic is mostly attributed to the promotion of drug uptake (7, 19, 41, 42), thereby being designed to remove the uptake barrier to combat antibiotic resistance as next-generation antibacterial agents (11). This is because intracellular antibiotic concentration is decisive for killing bacteria. Bacterial antibiotic resistance mechanisms include a decrease in membrane permeability, an increase in enzymatic degradation, an elevation in efflux pump, and modification of targets (43–45). The decrease of membrane permeability prevents the entrance of extracellular antibacterial agents, while the other three dampen the action of intracellular antibacterial agents. Increased membrane permeability can promote antibiotic uptake. When the promoting antibiotic uptake is large enough to exceed the amount dampened, these antibiotic resistance mechanisms that dampen the action of intracellular antibacterial agents will be overcome (11). Among the three mechanisms dampening intracellular antibacterial agents, the elevated efflux pump plays the most key role in MRSA (46). Thus, intracellular cefoperazone is measured in the presence or absence of exogenous valine to investigate whether the valine-promoted uptake overcomes the efflux pump in the present study. Exogenous valine promotes drug uptake, efflux, and hydrolysis, but the uptake is much greater than the efflux and hydrolysis. This leads to an increase of the intracellular antibiotic concentration by about 3.05 times. This finding not only explains the valine-potentiated killing efficacy by increasing the intracellular drug in MRSA2, but also indicates the potential for developing the combination of SCF with valine as the next drug to combat MRSA.

To understand mechanisms by which valine promotes drug uptake, reprogramming metabolic state and NTFD approaches are used to track the valine metabolic flux. This leads to the findings on PMF elevation and membrane permeability increase. The valine-induced PMF elevation and membrane permeability increase are responsible for the elevated uptake. The PMF elevation is attributed to the activation of the P cycle/the TCA cycle via the valine-succinate metabolic pathway to produce more NADH, while the membrane permeability increase results from lauric acid. Lauric acid is related to membrane permeability (31, 32). Significantly, the synergistic effect of the elevated PMF and increased membrane permeability leads to increased drug uptake. These findings explain why high killing efficacy is detected in the valine-induced potentiation.

PAE is one characteristic of pharmacodynamics, implying the transient suppression of bacterial magnification after antibiotic treatment and providing data regarding antibiotic activity. PEA allows antibiotic dosing regimens to be developed on a more scientific basis (28, 47). Thus, the increase of PEA can alter antibiotic dosages, especially important for new drugs that promote antibiotic uptake.

In summary, the comparison of the metabolic states between MRSA and MSSA isolates reveals a most repressed metabolism and identifies valine as the most crucial biomarker in MRSA isolates. Valine as a reprogramming metabolite reverses MRSA resistance to synergize SCF to effectively kill clinically isolated MRSA isolates *in vitro* and *in vivo*. Valine promotes MRSA to uptake SCF that overcomes the efflux via the elevated PMF and membrane permeability. It also extends the PAE of SCF. These results highlight the way tp utilize metabolic reprogramming to combat MRSA.

## MATERIALS AND METHODS

### Chemicals

Luria-Bertani (LB) medium and Mueller-Hinton broth (MHB) medium were purchased from Huankai Biotech Limited (Guangzhou, China). All antibiotics tested, including vancomycin (glycopeptides), cefoperazone sodium and sulbactam sodium (cephalosporins), daptomycin (cyclic lipopeptides), oxacillin (penicillins) were purchased from Sangon Biotech (BBI) Limited (Shanghai, China). Metabolome sample preparation and derivatization reagents, including methanol, pyridine, and methoxyamine hydrochloride, were purchased from Thermo Fisher Scientific.

### Bacterial strains and MIC tests

MSSA and MRSA isolates were from Zhongshan Hospital, Xiamen University, and were kept in collections of our laboratory. MSSA and MRSA isolates were stored in the refrigerator at −80°C (70% bacterial solution, 30% glycerin). These bacteria were cultured in MHB medium at 37°C and 200 rpm for 16 h. A microdilution broth susceptibility assay for bacteria was used, as recommended by The Clinical & Laboratory Standards Institute (CLSI) for the determination of the minimum inhibitory concentration. Serial twofold dilutions of antibiotic (from 120 to 0.05 μg/mL) were prepared in a 96-well plate (100 µL per well). Wells with no SCF were used as a positive growth control. A diluted bacterial suspension in MHB medium was added to each well to give a final concentration of 5 × 10^4^ colony-forming units (CFU)/mL, confirmed by viable counts. Wells without bacteria were used as a negative growth control. The plate was incubated for 16–20 h at 37°C and bacterial growth was visually assessed. The MIC was defined as the lowest antibiotic concentration without visible growth. At least three independent determinations were performed.

### Phenotypic characterization of the production of β-lactamase

The screening of CRE phenotypes and identification of metallo-β-lactamases (MBLs) was performed according to the mCIM/eCIM method recommended in the 2019 US CLSI guidelines. The saturated bacteria to be tested from the overnight incubation at 37°C were inoculated into two 3 mL tubes of TSB medium (1:2,000) and incubated at 37°C, 200 rpm for 4 h. The bacteria were used for mCIM and eCIM assays, respectively (30 µL of 0.5 mol/L EDTA was added to this tube). At the same time, *E. coli* ATCC25922 was used as the test strain for testing enzyme production, which was cultured overnight to saturation, diluted with saline to OD_625_ = 0.08–0.13, and 100 µL was taken and coated on the MHB plate. After the plate was dry, the drug-sensitive paper pieces from two tubes of TSB broth of the same strain to be tested were taken out and pasted on the same MHB plate, ensuring that the distance between the centers of the paper pieces was not less than 24 mm, and that the distance between the paper pieces and the edges was not less than 15 mm.The size of the circle of inhibition was measured after inverted incubation at 37°C for 20 h. When 10 µg of meropenem was detected in the plate, the size of the circle was determined. When the diameter of the inhibition circle formed by the 10 µg meropenem paper sheet is greater than or equal to 19 mm, then it does not produce CRE; less than 19 mm but greater than 15 mm or the inhibition circle has scattered colonies, then it is judged as intermediate state; less than or equal to 15 mm, then it is judged as CRE production. When it is judged to produce CRE, then it can be further judged whether to produce metalloenzyme, otherwise no judgment. The judgment method is compared with the use of meropenem alone, meropenem paper combined with EDTA together with the circle of inhibition diameter is greater than or equal to 5 mm, it is judged to produce MBL.

### Phenotypic characterization of ESBLs-producing and AmpC enzymes

The bacterial solution was diluted using the above method and 100 µL was taken and spread evenly on the MHB plate. After the plates were dried, 30 µg ceftazidime (CAZ) and 30 µg ceftazidime–10 μg clavulanic acid (CCV) combined paper sheets were pasted on the same MHB plate, and 30 µg cefotaxime (CTX) and 30 µg cefotaxime–100 μg clavulanic acid (CTC) combined paper sheets were pasted on the same plate. The plates were incubated in an inverted incubator at 37°C for 16 h to measure the diameter of the circle of inhibition and photographed for storage. When the bacteria produce ESBLs, the circle of inhibition formed by CCV or CTC combined paper sheet will be larger than that formed by CAZ or CTX paper sheet alone due to the inhibiting effect of the inhibitor on ESBLs, if the diameter of the circle of inhibition formed by CCV or CTC when used together is greater than or equal to the diameter of the circle of inhibition formed by the corresponding single piece of paper, it will be judged as an ESBLs-producing positive strain. AmpC enzyme was detected according to the K-B paper diffusion method recommended by CLSI 2021. The experimental operation was the same as that of ESBLs detection, and the strain was suspected of producing AmpCase when the diameter of the inhibition circle was less than or equal to 18 mm in 30 µg cefoxitin (FOX)-resistant paper sheet.

### GC-MS sample preparation and analysis

GC-MS analysis was performed, as previously described (7). MSSA and MRSA strains were incubated in LB medium overnight at 37°C, collected by centrifugation with 200 rpm, diluted into an OD_600_ nm of 1.0 in M9 medium, and cultured for 10 h. These cells with 10 mL at OD_600_ of 1.0 were collected, immediately quenched with precooled methanol (HPLC grade) and sonicated for 15 min (200 W total power with 35% output, 2 s pulse, 3 s pause over ice) for intracellular components including metabolites, followed by centrifugation at 4°C and 12,000 rpm for 10 min for supernatants. The supernatants were isolated and 10 µL of ribitol (0.1 mg/mL, Sigma) was added as an internal standard, and dried by a Labconco (USA) vacuum centrifuge dryer at a temperature of 37°C. The dried extracts were incubated with 80 µL of methoxime-pyridine hydrochloride (20 mg/mL, Sigma-Aldrich) in pyridine (Sigma-Aldrich) for 3 h at 37°C, and then metabolite derivatization was done with 80 µL of n-methyl-n -trimethylsilyl trifluoroacetamide (MSTFA, Sigma-Aldrich) for another 45 min. Samples were centrifuged at 12,000 rpm for 10 min, and the supernatant was transferred into new tubes to untargeted gas chromatography-mass spectrometry (GC-MS) analysis (Agilent).

A modified two-stage technique was utilized to perform GC-MS analysis combining Agilent 7890A GC with the Agilent 5975C VL MSD detector (Agilent Technologies) (7). Approximately 1 µL of samples was infused into a 30-m long and 250-mm wide (i.d. with 0.25 mm DBS-MS column) cylinder. The GC oven’s initial temperature was maintained at 85°C for 5 min, and then increased to 270°C at a rate of 15 °C/min and maintained for 5 min. Helium was utilized as the carrier gas, with flow was 1 mL/min. The MS could operate between 50 and 600 *m/z*. Three biological repeats and two technical repeats were used.

Initial peak detection and mass spectral deconvolution were analyzed using XCalibur software (Thermo Fisher version 2.1). The metabolites were identified from the National Institute of Standards and Technology (NIST) database and the NIST MS Search 2.0 program. The peak areas of these metabolites were corrected using the internal standard (ribitol). Then, these metabolites were analyzed as follows: identification of metabolites that had a statistically significant difference (*P* value < 0.05) using IBM SPSS Statistics 19 software; Cluster analysis using the R software (R × 64 3.6.1); principal component analysis and S-plot [|*P* (1)| < 0.05 and |*P*(corr)1| < 0.5 are considered biomarkers] analysis using SIMCA-P + 12.0 software (version 12; Umetrics, Umea, Sweden); the control and experimental group *Z*-values were analyzed to determine whether the metabolism of the metabolites was upregulated or downregulated; pathway enrichment of SPSS-calculated discrepant substances at website (http://www.metaboanalyst.ca/).

### Antibiotic bactericidal assays

Antibiotic bactericidal assay was carried out, as previously described (6). In brief, three to four colonies were selected from the plate and cultured in a test tube containing 5 mL and cultured at 37°C for 16 h. After centrifugation at 8,000 rpm for 5 min, samples were washed two times with 15 mL sterile saline and re-suspended in M9 minimal media supplemented with 10 mM acetate and 100 mM CaCl_2_. Subsequently, the bacterial population was diluted with M9 medium to approximately 2.5 × 10^7^ CFU/mL and the desired metabolite (valine) and antibiotics were added. These mixtures were incubated at 37°C for 10 h. The use of M9 medium offers us the advantage to test the effect of desired metabolites on antibiotic-resistant strains in a reliable way, by which potential confounding factors would not be present as in more complex medium. After incubation at 37°C, 200 rpm for 10 h, 100 µL of the bacterial culture was taken for 10-fold dilution, followed by 5 µL of the distribution on an LB plate. The plates were cultured at 37°C for 16–22 h. Only those dilutions yielding 20–200 colonies were enumerated to calculate CFU. Percent survival was determined by dividing the CFU obtained from a treated sample by the CFU obtained from the control.

### Membrane potential measurement

PMF was measured as previously described using the BacLight bacterial membrane potential kit (Invitrogen) (48). Bacteria were collected by centrifugation and re-suspended in 1 mL of saline. The cells were labeled with 10 µL of 3 mM DiOC_2_ and incubated for 30 min at 37°C. Aliquots with 1 mL of samples were analyzed using a FACSCalibur flow cytometer (Becton Dickinson, San Jose, CA, USA) under a 488 nm excitation wavelength. Forward versus side scatter and gated of samples were observed before the acquisition of data. The membrane potential was calculated by formula of membrane potential: log (10^3/2^ × [red fluorescence/green fluorescence]). Triplicate repeats were carried out.

### ATP determination

Bacterial suspensions (OD_600_ = 0.6) were collected and resuspended in saline solution. The samples were diluted into colonies of 10^7^ CFU/mL and dispensed in 50 µL in a 96-well plate. The samples were then mixed with 50 µL of pre-equilibrated BacTiter-Glo reagent (Promega, USA) for 5 min, and the luminescence was measured using a Victor X5 multimode plate reader.

### Determination of NADH

The NADH was measured with the EnzyChrom NADH assay kit (BioAssay Systems). Briefly, MSSA, MRSA, and MRSA + valine were incubated in M9 medium for 10 h. The cells were harvested and diluted to an OD_600_ of 1.0. Then, 2 mL of the cells was collected and washed three times with sterile saline. The resulting cells were resuspended in NADH extraction buffer vortex for 15 s. Then, the samples were incubated in 60°C water for 5 min. Assay buffer and opposite extraction exaction buffer were added. After vortex for 10 s and centrifugation at 12,000 × *g* for 5 min, the supernatant was used for measurement according to the manufacturer’s instructions of the NADH assay kit.

### Quantification of enzyme activity

The quantification of pyruvate dehydrogenase (PDH), α-ketoglutarate dehydrogenase (KGDH), succinate dehydrogenase (SDH), and malate dehydrogenase (MDH) activities was performed as described previously with some modifications (29). In brief, the MSSA, MRSA, and MRSA + valine were harvested and resuspended in M9 medium to OD_600_ =  1.0 (logarithmic phase). Samples with 30 mL were collected by centrifugation at 8,000 rpm for 5 min. The cells were re-suspended in phosphate-buffered saline and broken down by sonication for 15 min (650 W total power with 35% output, 2 s pulse, 3 s pause over ice), followed by centrifugation at 12,000 rpm for 10 min. Protein concentration was determined using a BCA protein assay kit (Beyotime Biotechnology, P0009). Then, 150 µg of proteins was spectrophotometrically monitored for reduction of MTT at 562 nm to characterize enzyme activity.

### Quantification of intracellular cefoperazone content

The content of bacterial intracellular cefoperazone was assayed by cefoperazone ELISA test kit manual. Fifty milliliters of MRSA with an OD_600_ of 0.2 was placed in M9 medium containing the desired valine and cefoperazone-sulbactam and incubated at 37°C for 10 h. The cells were collected and washed three times with cefoperazone extraction buffer and then resuspended with the cefoperazone extraction buffer and adjusted to OD_600_ of 1.0. An aliquot of 10 mL was collected and resuspended with 0.5 mL cefoperazone extraction buffer and transferred into a 1.5 mL centrifuge tube. The cells were disrupted by sonic oscillation for 10 min (650 W total power with 35% output, 2 s pulse, 3 s pause over ice). The resulting supernatant was collected for the detection of cefoperazone under the guidelines of the ELISA gentamicin detection kit.

### Efflux kinetic

The efflux kinetics of MRSA2 was determined at different extracellular antibiotic concentrations (7). The efflux rate *V*_e_ was calculated using the Michaelis-Menten equation {*V*_e_ = *V*_emax_ × (*C*_p_)*h*/[(*K*_0.5_)*h* + (*C*_p_)*h*]}, where *V*_emax_ is the maximal efflux rate of 0.37 nmol/mg/), *K*_0.5_ is the half-maximal flow rate drug concentration of 0.94 µM, and *h* is the Hill coefficient of 0.23. *C*_p_ was calculated as the intracellular cefoperazone content determined above. In the absence of exogenous valine, *V*_in_ was calculated using the formula *V*_in_ = *P* × *A* × (*C*_0_ − *C*_p_). The *C*_0_ value is the concentration of cefoperazone to the MRSA2 at 1 MIC value of 40 µg/mL (equivalent to 0.03 mM). When valine is added, *V*_in_ can be calculated as *V*_in_ = *P* × *A* × (*C*_p_ without valine/*C*_p_ with valine) × (*C*_0_ − *C*_p_). In the above equation, *P* is the osmotic coefficient (0.28 × 10^−5^ cm/s), and *A* is the cell surface area (constant value, 10^3^ cm^2^/mg) (49).

### Post-antibiotic effect

PAE experiment was performed against the same isolates used in time-kill study. The procedure of the experiment is as previously described with some modifications (50). Bacteria at logarithmic growth phase were exposed to SCF or SCF + valine in Mueller Hinton broth for 1 h incubation at 37°C. The concentrations of SCF used were at multiple MIC (0.5 MIC, 1.0 MIC, and 2.0 MIC) and the valine concentration was 5 mM. SCF or SCF + valine was removed by centrifugation at 8,000 × *g* for 3 min and then washed three times. Bacterial pellet was re-suspended in 4.5 mL M9 medium and incubated at 37°C for 12 h. Viable colony count was performed at time 0 (immediately after addition of SCF), immediately after SCF removal and every 2 h after SCF removal for up to 14 h. Control growth was simultaneously conducted without SCF addition. PAE was determined using formula: PAE = *T* – *C*, where *T* is the time required for treated culture colony count to increase by 10-fold from the count obtained immediately after SCF removal and *C* is the corresponding time required for control culture. Three independent experiments were conducted for each isolate to ensure reproducibility and variability.

### Animal studies

Six-week-old male BALB/c mice with weight of 20 ± 1 g were obtained from the Laboratory Animal Center of Sun Yat-sen University (Guangzhou, China), were acclimatized for 1 week, and conformed to institutional animal care and use policies. All animals were used.

### Valine-enabled killing of MRSA by SCF in a mouse systemic infection model

Valine combined with SCF was tested against clinical isolate MRSA2 in a mouse systemic infection assay to assess its *in vivo* bioavailability. The systemic infection included mouse survival and bacterial CFU counting of mouse organs. For survival, male BALB/c mice (10 per group) were intraperitoneally infected with 0.5 mL of bacterial suspension (2.5 × 10^7^). At 1 h later, mice were treated with saline solution, SCF, valine, or SCF + valine using intravenous injection. Survival is observed 7 days after infection. For CFU counting, male BALB/c mice (four per group) were intravenously infected with 0.5 mL of bacterial suspension (2.5 × 10^6^ CFU) and treated with saline solution, SCF, valine, or SCF + valine in an hour later. Spleen, kidney, and liver were collected at 24 h. The three organs were homogenized for spot plate counting (CFU/g).

### Valine-enabled killing of MRSA by SCF in a mouse thigh infection model

Valine combined with SCF was tested against MRSA2 in a neutropenic mouse thigh infection model. Male balb/C mice were rendered neutropenic by cyclophosphamide (two consecutive doses of 150 and 100 mg/kg delivered on 4 days and 1 day, respectively, before infection) (51). Bacteria were resuspended in saline solution, adjusted to an *A*_600_ (OD_600_) of 0.1, and a 0.1 mL inoculum (2.8 × 10^5^ CFU per mouse) injected into the right thighs of mice. At 2 h post-infection, mice (four mice per group) received intravenous treatment with saline solution, SCF (200 mg/kg), valine (5 mM), or SCF + valine. At 26 h post-infection, mice were euthanized by CO_2_ inhalation. The right thighs were aseptically removed, weighed, homogenized, serially diluted, and plated on LB for CFU titers.

### Isotope tracing analysis

To explore the metabolic flow of 13^C^ valine in MRSA, an equal mixture of unlabeled and [13 C^5^]-labeled valine was added to MRSA2 cells. The incubated bacteria were washed and adjusted to OD_600_ to 1.0. Subsequently, 10 mL of cell suspension was collected by centrifugation at 5,000 rpm and immediately treated with 1 mL of cold methanol (HPLC grade, Thermo Fisher Scientific Company) to stop bacterial metabolic activity. As an internal standard, 10 µL of ribitol solution (0.1 mg/mL) was added to the cell suspension, which was sonicated for 20 min at 200 W operating power. The sonicated fractions were centrifuged at 4°C, 12,000 rpm for 10 min. The supernatant was transferred to a vacuum centrifuge dryer (Labconco, USA) at 37°C and methanol was removed by evaporation. The dried sample was added with 80 µL of 20 mg/mL methoxime pyridine hydrochloride (Sigma-Aldrich), dissolved by ultrasonication and reacted at 37°C for 3 h. Subsequently, 80 µL of N-methyl-N-trimethylsilyltrifluoroacetamide (MSTFA, Sigma-Aldrich) was added, which was carried out at 37°C for 45 min. GC- MS data were obtained using an Agilent 7890A gas chromatograph equipped with an Agilent 5975C VL MSD detector (Agilent Technologies). Aliquot with 1 µL of sample was injected into a 30 m × 250 mm (inner diameter), 0.25 mm DBS-MS column. The gas chromatography was held at an initial temperature of 85°C for 5 min, then ramped up to 270°C at a rate of 15 °C/min, and held for 5 min. Helium was used as the carrier gas at a flow rate of 1 mL/min. The MS was operated in the range of 50–600 *m/z*. Three biological and two technical repeat sequences were prepared for each sample. Ion fragmentation spectra were analyzed, using the 2008 version of the NIST mass spectral library. Graphs were created using GraphPad Prism 8.0.

### Membrane permeability

The bacteria were resuspended with M9 medium and adjusted to 0.2 of OD_600_. A desired concentration of metabolites was added. The incubation was placed in a shaker at 37°C, 200 rpm for 10 h. An aliquot of 100 µL sample was mixed with 900 µL M9 medium and then 2 µL of 2.5 mM SYTO9 dye was added. Samples were incubated at 37°C, 200 rpm shaker for 30 min, followed by detection of green light intensity by flow cytometry.

### Statistical analysis

Statistics are carried out and graphed using Prism v7 (GraphPad). After testing for normality, a two-tailed paired Student’s *t* test was used for all pairwise statistical comparisons unless otherwise noted. Differences are significant at *P* < 0.05 and highly significant at *P* < 0.01.

## Data Availability

The data of the metabolome of valine have been uploaded to MetaboLights under number MTBLS11540.

## SUPPLEMENTAL MATERIAL

Supplemental material msystems.01244-24-s0001.docx Tables S1 to S3; Fig. S1 to S5.

ASM does not own the copyrights to Supplemental Material that may be linked to, or accessed through, an article. The authors have granted ASM a non-exclusive, world-wide license to publish the Supplemental Material files. Please contact the corresponding author directly for reuse.
