# Supplementary material for: Valine potentiates cefoperazone-sulbactam to kill methicillin-resistant Staphylococcus aureus
Source: mSystems. 2024 Dec 18;10(1):e01244-24. doi: 10.1128/msystems.01244-24 (PMC11748551; doi:10.1128/msystems.01244-24)
Supplement: Supplemental material — Tables S1 to S3; Fig. S1 to S5. [file msystems.01244-24-s0001.docx]

**Supplementary materials**

**Supplementary Table 1. Primers used for PCR**

| Gene | Primer | Primer sequence | length (bp) |
| --- | --- | --- | --- |
| *femB* | Forward | 5'-TTACAGAGTTAACTGTTACC-3' | 651 |
|  | Reverse | 5'-ATACAAATCCAGCACGCTCT-3' |  |
| *pvl* | Forward | 5'-ATCATTAGGTAAAATGTCTGGACATGATCCA-3' | 433 |
|  | Reverse | 5'-GCATCAAGTGTATTGGATAGCAAAAGC-3' |  |
| *mecA* | Forward | 5'-CCAATTCCACATTGTTTCGGTCTAA-3' | 310 |
|  | Reverse | 5'-GTGCGCCATGTTCGTAGAAACG-3' |  |

**Supplementary Table 2. MIC measurement of MRSA and MSSA strains**


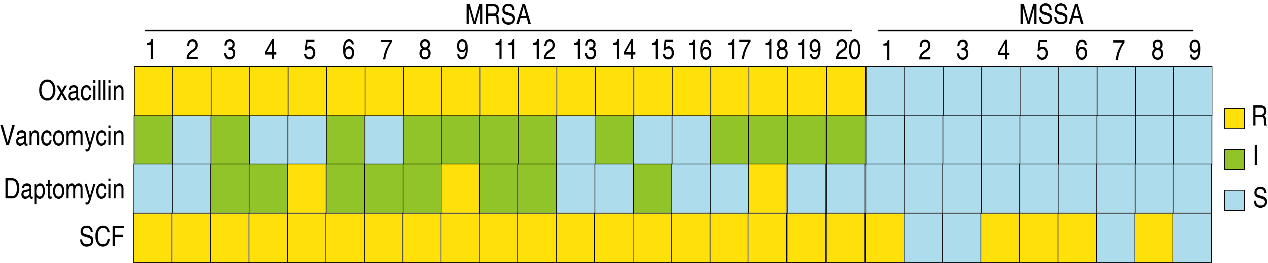


Note: Antibiotics refer to the 2022 American Clinical and Laboratory Standards Association (CLSI) standards, and the remaining 2 types of CLSI without standards are determined to be resistant to this antibiotic according to the conventional MIC standards, that is, the MIC of resistant bacteria/sensitive bacteria ≥ 4.

**Supplementary Table 3. Identification of β-Lactamases**


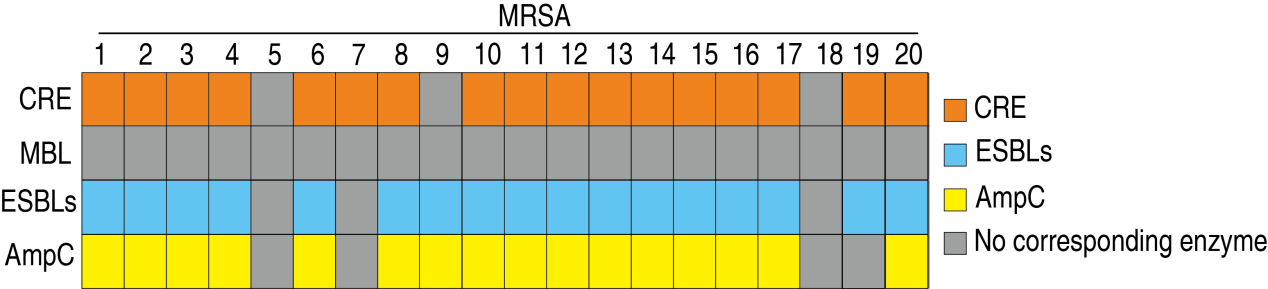


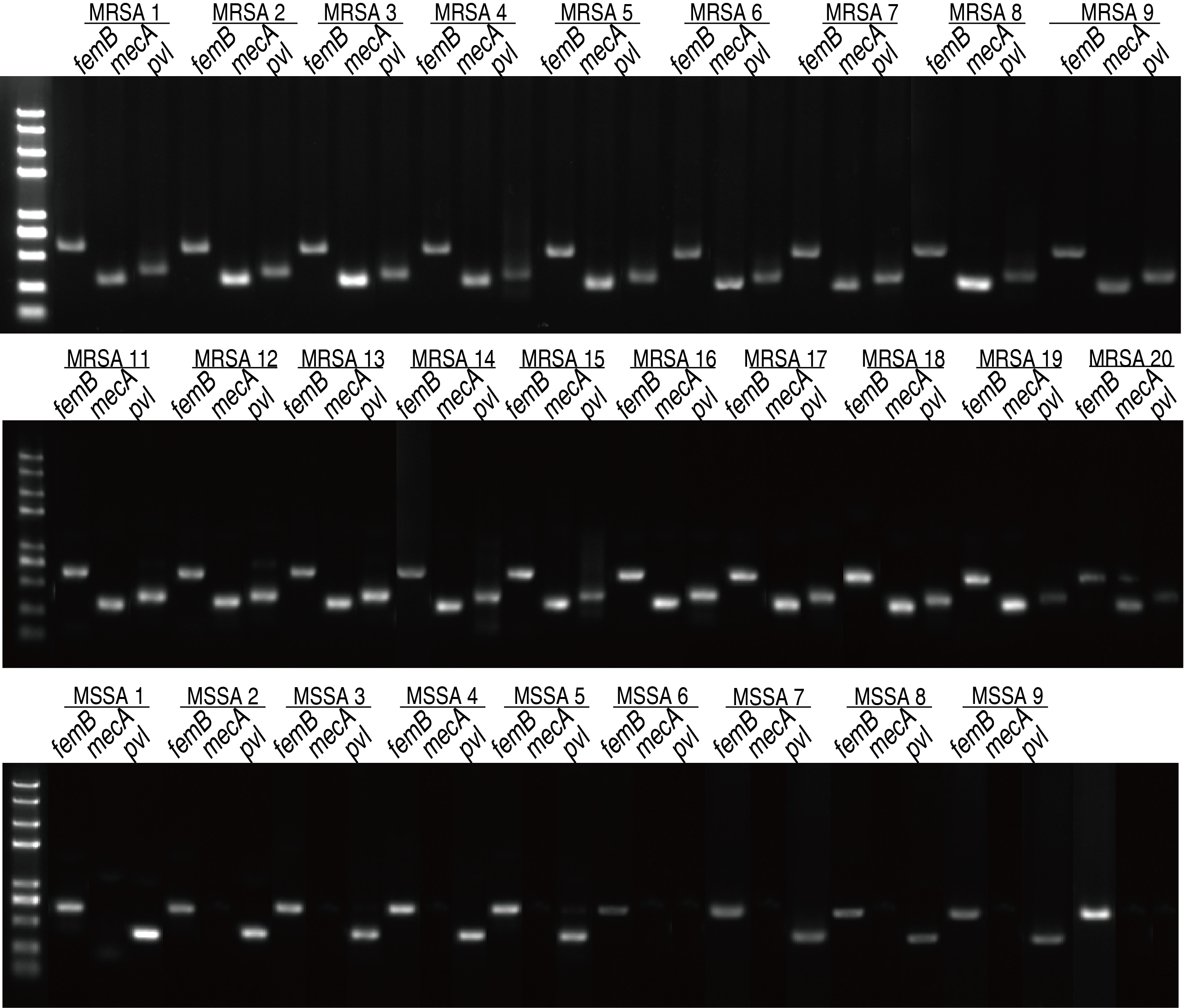


**Figure S1. PCR detection for *femB*, *mecA*, and *pvl*.**


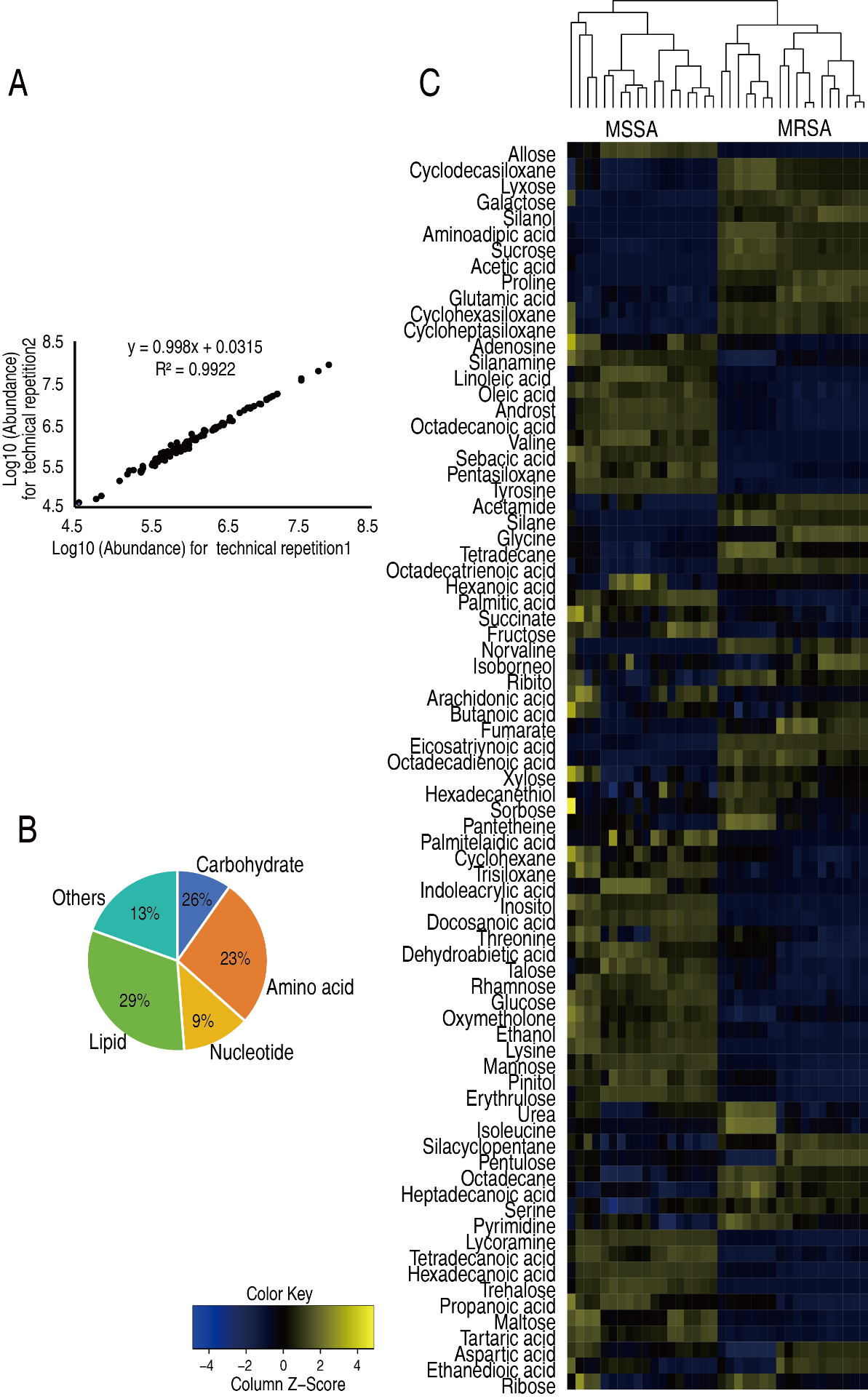


**Figure S2. Metabolic profiles of MSSA and MRSA**. (A) Technical reproducibility analysis of metabolomics. (B) Detected metabolite classes. (C) An overall metabolite cluster map.


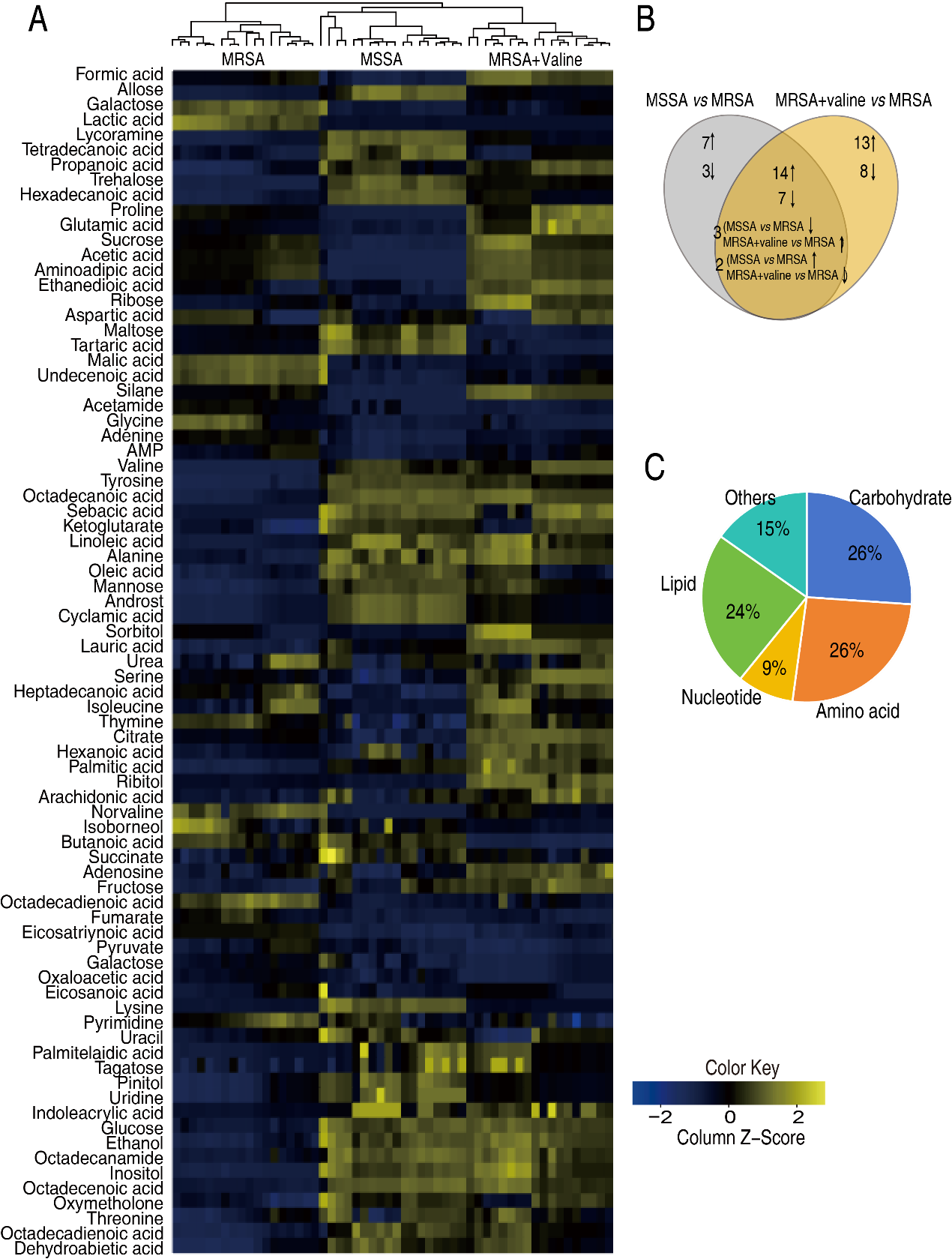
**Figure S3. Metabolic profiles of MRSA with and without exogenous valine and MSSA.** (A) An overall metabolite cluster map. (B) Venn diagram showing the overlapping and unique differential metabolites between MSSA and MRSA + valine compared to MRSA. Upward and downward arrows indicate increase and decrease, respectively, at metabolite abundance compared to the control group. (C) Detected metabolite classes.

**
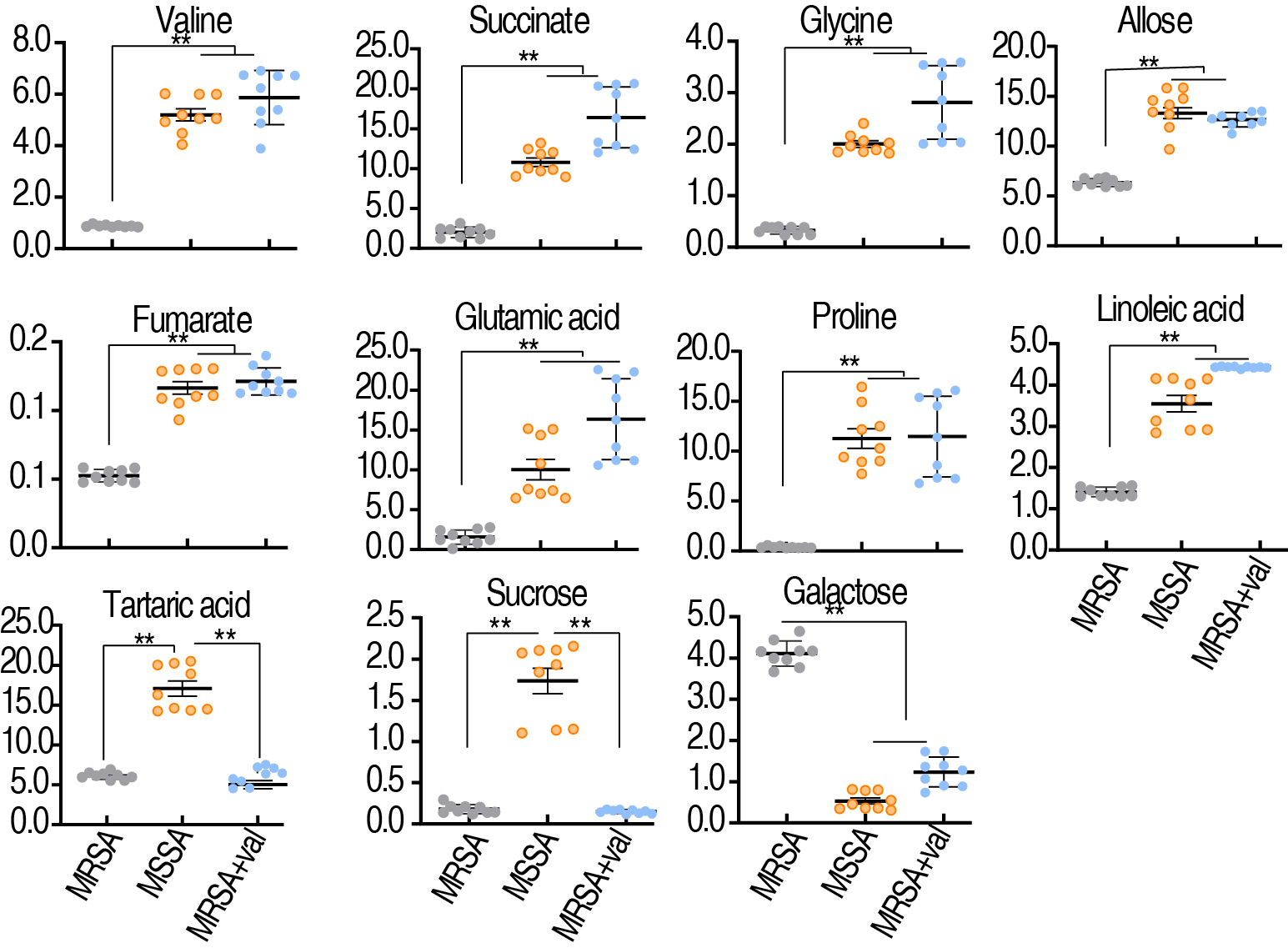
**

**Figure S4. Scatter diagram of 11 biomarkers i**n **the valine-reprogrammed metabolomes.**


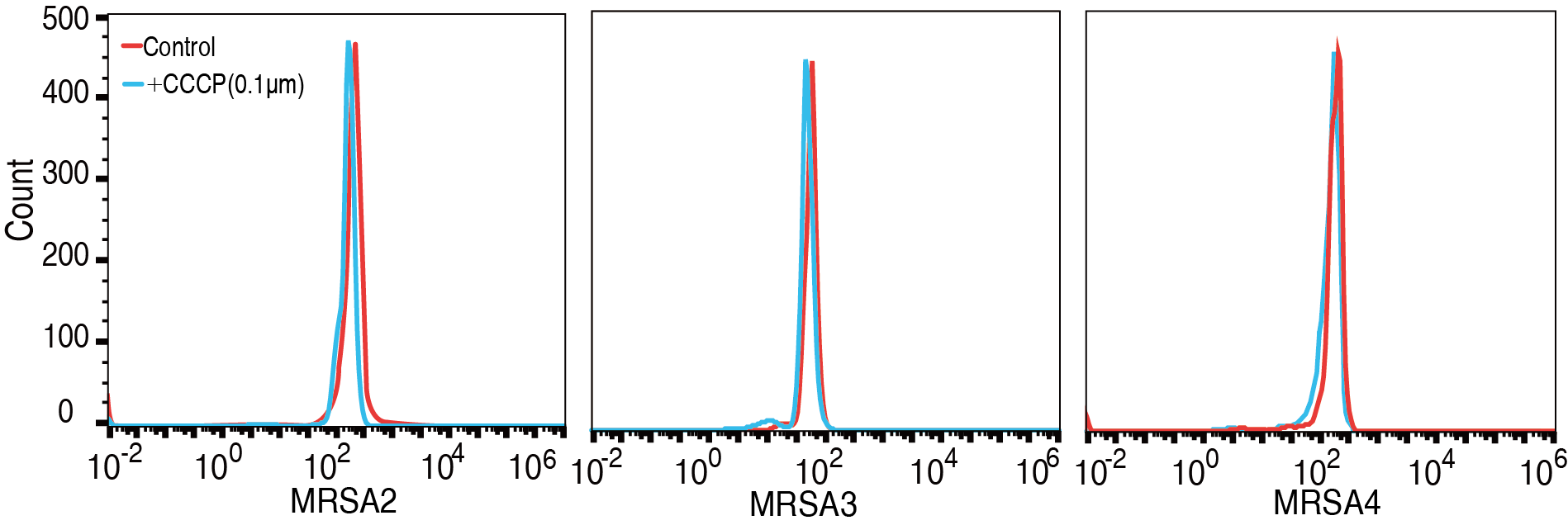


**Figure S5. Representatives of maps showing effect of CCCP or valine on membrane permeability in the indicated MRSA isolates**
